# The GhWRKY65–*GhAOC3A* Module Enhances the Chilling Tolerance in Cotton by Regulating Jasmonic Acid Accumulation

**DOI:** 10.3390/plants15213417

**Published:** 2026-11-03

**Authors:** Gang Chen, Jianting Feng, Haohua Chen, Shiyan Cui, Xuechi Li, Rui Tang, Manhong Wang, Lingling Chen, Shandang Shi, Fei Wang, Hongbin Li

**Affiliations:** 1 Key Laboratory of Oasis Town and Mountain-Basin System Ecology of Xinjiang Production and Construction Corps, Key Laboratory of Xinjiang Phytomedicine Resource and Utilization of Ministry of Education, College of Life Sciences, Shihezi University, Shihezi 832003, China; cg_sdjn@163.com (G.C.);; 2 Department of Poultry Science, Mississippi State University, Starkville, MS 39762, USA

**Keywords:** upland cotton, allene oxide cyclase (AOC), chilling stress, WRKY transcription factor, jasmonic acid, regulatory module

## Abstract

Allene oxide cyclase (AOC) is an integral enzyme in jasmonic acid (JA) biosynthesis and a key regulator of plant stress responses. However, the regulatory mechanisms and enzymatic activities of cotton *AOC* under stress responses remain poorly understood. In our research, using BLASTP and conserved domain validation, we identified 68 *AOC* gene members across 10 *Gossypium* species. Phylogenetic, gene-structure, and syntenic analyses showed high evolutionary conservation of *AOC* genes, accompanied by lineage-specific expansion. Promoter sequence analysis indicated that the *AOCs* contained abundant *cis*-elements associated with hormone signaling, abiotic-stress response, and transcription factor (TF) regulation. Expression profiling revealed tissue-specific and stress-responsive expression patterns among *GhAOC* genes, with *GhAOC3A* showing the most pronounced upregulation under chilling stress. GhAOC3A was identified as a chloroplast-localized protein based on the subcellular localization assay. *GhAOC3A* overexpression enhanced cotton chilling tolerance with elevated JA accumulation, stronger antioxidant defense, stable osmotic homeostasis, and retained chlorophyll content, and VIGS results provided supportive evidence for the function of *GhAOC3A* in chilling tolerance. Among the predicted TFs that could regulate the expression of *GhAOC3A*, GhWRKY65 was further identified and confirmed to bind to the W-box element and activate *GhAOC3A* transcription. Overexpression of *GhWRKY65* significantly enhanced cotton chilling tolerance, as well as elevated *GhAOC3A* expression and increased JA accumulation. Conversely, silencing *GhWRKY65* compromised cotton chilling tolerance. Collectively, these findings suggest that the GhWRKY65-*GhAOC3A* regulatory module positively modulates cotton chilling tolerance and JA levels. Our study provides insights into the transcriptional network associated with plant stress response and offers effective candidates for breeding chilling-tolerant cotton materials.

## 1. Introduction

Cotton (*Gossypium* spp.) is a globally crucial commercial crop that produces and provides essential natural fibers that are core raw materials for the textile and related industries [1]. Originating from warm tropical and subtropical regions where cold events rarely occur, cotton exhibits poor capacity to acclimate effectively to low-temperature environments [2]. The expansion of cotton cultivation to higher latitudes is a pivotal strategy for increasing global production; however, this approach is severely constrained by cold stress—a major abiotic factor that considerably suppresses cotton growth and reduces crop productivity [3]. Specifically, cold stress not only disrupts seedling establishment and development but also triggers widespread wilting and mortality, ultimately reducing fiber quality and yield [4]. Under cold stress, cotton seedlings develop multiple physiological injuries, including leaf chlorosis, reduced photosynthetic efficiency, and disrupted reactive oxygen species (ROS) homeostasis [5]. Collectively, these changes suppress cotton growth and development.

Over long-term evolution, plants have developed specialized hormone-signaling networks to cope with cold stress. Jasmonic acid (JA) serves as a pivotal phytohormone that mediates stress responses and regulates plant adaptations to cold environments [6]. Previous studies have demonstrated that cold stimulus rapidly activates JA biosynthesis and downstream signaling cascades, thereby enhancing cold tolerance by activating antioxidant systems, promoting osmolyte accumulation, and upregulating cold-stress-responsive genes [7]. Exogenous JA treatment was demonstrated to improve the cold tolerance of *Prunus persica* fruits under cold storage conditions [8]. JA biosynthesis predominantly takes place in two subcellular compartments: chloroplasts and peroxisomes [9]. It starts with α-linolenic acid, followed by sequential catalysis by lipoxygenase (LOX), allene oxide synthase (AOS), allene oxide cyclase (AOC), 12-oxophytodienoate reductase (OPR), and a set of β-oxidation-related enzymes to generate JA [10,11]. Among these enzymes, AOC is the key component of JA biosynthesis, catalyzing the cyclization of unstable allene oxide intermediates to generate biologically active 12-oxophytodienoic acid (12-OPDA). This step determines JA stereochemistry and governs its subsequent signaling potency [12,13]. Nevertheless, these findings are mainly derived from other plants [14]. Little is known about the function of cotton *AOC* and the JA-dependent regulatory mechanisms underlying cold stress. Therefore, studying cotton AOC and its associated molecular mechanisms is essential to understand JA-mediated cold adaptation in cotton.

Copy numbers of allene oxide cyclase (AOC) homologs display substantial interspecific variation: *Oryza sativa*, *Sorghum bicolor*, and *Solanum lycopersicum* each harbor a single *AOC* gene, while *Glycine max*, *Populus trichocarpa*, *Arabidopsis thaliana*, *Citrullus lanatus*, and *Zea mays* possess 6, 3, 4, 2, and 2 homologs, respectively [15,16]. Across both biotic and abiotic stresses, *AOC* family members demonstrate functional divergence and are extensively implicated in regulating plant stress adaptation [17]. *GmAOC3* is a viable target for insect-resistant molecular breeding, and its overexpression enhances soybean resistance to generalist leaf-chewing larvae [18]. *ScAOC1* responds to both smut infection and abiotic stresses including chilling, salinity, and drought, thereby improving overall stress tolerance via activation of defense-related signaling pathways [19]. *ClAOC2* from *Citrullus lanatus* primarily mediates red light-activated defense against root-knot nematodes and stress responses [15]. Loss of function of *O. sativa AOC* induces abnormal floral organ development associated with a sharp drop in the levels of endogenous JA and compromised resistance to *Magnaporthe oryzae* [20]. Wheat *TaAOC1* augments salt tolerance by promoting JA accumulation and optimizing root system architecture [21]. In *Gossypium hirsutum*, *GhAOC1* improves plant survival, biomass accumulation, and photosynthetic performance and alleviates lipid peroxidation and membrane damage [22]. Conversely, suppression of *GhAOC2* disrupts JA homeostasis under heat stress, resulting in overaccumulation of ROS in anthers and abnormal tapetum degradation, ultimately inducing male sterility [23].

Transcription factors (TFs) regulate diverse metabolic and biosynthetic pathways that support the coordinated progression of plant life processes [24]. Diverse TF families establish multilayered regulatory networks that integrate endogenous hormonal cues and environmental signals [25]. They maintain proper plant development and metabolic homeostasis and ensure orderly life-cycle completion through precise repression or activation of downstream target genes [26]. Among these TFs, WRKY family members serve as pivotal orchestrators of plant defense mechanisms against both abiotic and biotic stresses. SlWRKY75 enhances drought tolerance throughout the growth cycle by transcriptionally repressing *SlARF5* and *SlTRY* [27]. MdWRKY75 directly targets the promoters of *heat shock factor* (*HSF*) genes to activate transcription under heat stress, thereby positively regulating basal thermotolerance [28]. *GmWRKY21* improves aluminum stress tolerance by promoting proline accumulation and mitigating membrane lipid peroxidation [17]. In cotton, increasing evidence highlights the diverse functions of *WRKY* genes in regulating stress modulation and development. GhWRKY1-like binds to the promoters of key ABA biosynthetic genes (*NCED2*/*5*/*6*/*9*), promoting endogenous ABA accumulation and thereby enhancing drought tolerance [29]. GhWRKY70 provides Verticillium wilt resistance in cotton through transcriptional activation of *GhAOS1* expression and JA pathway signaling [30]. However, it remains unclear whether *WRKY* modulates JA metabolism through *AOC* genes to facilitate chilling tolerance in cotton.

Although extensive reports show that *AOCs* mediate stress in many plants, the regulatory mechanisms and biological functions of cotton *AOCs* in cold-stress responses remain poorly understood. In our research, we first performed genome-wide identification and characterization of *AOC* genes across 10 *Gossypium* species and comprehensively analyzed their molecular features. Additionally, we further analyzed the expression patterns of upland cotton *AOC* genes under multiple abiotic stresses and in different tissues. Among these AOC members, *GhAOC3A* exhibited the strongest induction under low temperature, and *GhAOC3A* expression was positively correlated with chilling resistance. Moreover, we hypothesized that GhWRKY65 could directly bind to and activate the *GhAOC3A* promoter to elevate JA accumulation, thereby establishing a novel GhWRKY65-*GhAOC3A* regulatory module implicated in cotton chilling tolerance. To verify this hypothesis, we performed the molecular and physiological assays described above.

## 2. Results

### 2.1. Identification and Chromosomal Localization of the AOC Genes

To systematically identify members of the *AOC* gene family in ten *Gossypium* species, we performed BLASTP searches against their respective genomes. Four well-characterized *AOC* homolog protein sequences from *A. thaliana* were used as queries. This genome-wide analysis led to the identification of 68 *AOC* gene members. Based on their chromosomal locations, all *AOCs* were systematically named, showing a clear symmetric distribution between the A and D subgenomes of allotetraploid cotton species (Figure S1). Notably, the distribution patterns on chromosomes A08 and D08 were highly conserved, with genes relatively dense. By contrast, only one *AOC* gene was identified on chromosomes A12 and D12 in all tetraploid species, with a single gene on each. In diploid *Gossypium* species, *AOC* genes of *G. arboreum* and *G. herbaceum* were localized to chromosomes 08 and 12, displaying an arrangement similar to that of *AOC* genes in the A-subgenome of tetraploid cotton. In contrast, D-genome diploid species exhibited substantial interspecific variation in the chromosomal localization of *AOC* genes. *G. raimondii* harbored *AOCs* on chromosomes 04 and 08, *G. turneri* harbored *AOCs* on chromosomes 08 and 12, and *G. thurberi* possessed only an *AOC* gene on chromosome 02. The chromosomal distribution of *AOC* family members in *Gossypium* species exhibited both highly conserved core loci and distinct species-specific patterns across divergent genomes.

Physicochemical properties of the 68 identified AOC proteins were further characterized, revealing substantial interspecific variation in sequence length, net charge, and protein stability (Table S1), with the amino acid length ranging from 167 aa (GtAOC4A) to 314 aa (GhAOC4A), predicted molecular weight spanning from 18.63 kDa (GtAOC4A) to 35.26 kDa (GhAOC4A), theoretical isoelectric point (pI) extending from 5.85 (GdAOC3D) to 9.54 (GtAOC4A), and aliphatic index ranging from 72.85 (GtuAOC5) to 87.7 (GbAOC3A). The majority of AOCs had instability indices higher than 40, and they were projected to be hydrophilic proteins.

### 2.2. Phylogenetic Relationship and Structural Conservation of the AOC Genes in Cotton

**Figure 1:**
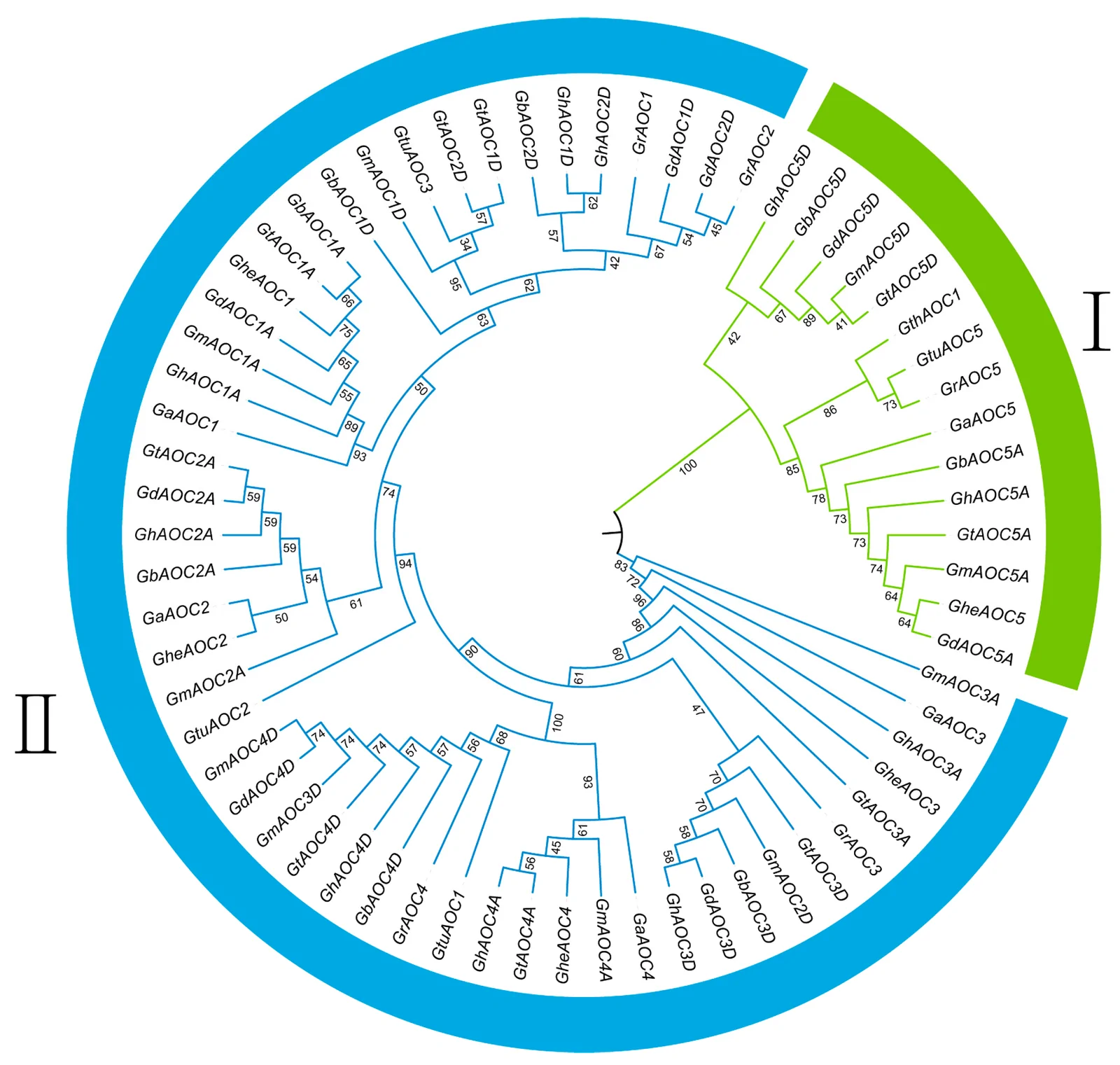
Phylogenetic tree of the *AOC* family genes in 10 *Gossypium* species. The phylogenetic tree was constructed with Neighbor-Joining (NJ) method. Varying colors signify distinct evolutionary clades. *Gossypium arboreum* (Ga), *G. herbaceum* (Ghe), *G. raimondii* (Gr), *G. thurberi* (Gth), *G. turneri* (Gtu), *G. darwinii* (Gd), *G. mustelinum* (Gm), *G. tomentosum* (Gt), *G. barbadense* (Gb), *G. hirsutum* (Gh).

A phylogenetic analysis of the 68 *AOCs* across 10 *Gossypium* species revealed that these *AOCs* clustered into two major clades. Clade I represented a monophyletic group encompassing all *AOC5*-type genes that were evolutionarily distinct from other subtypes. Clade II included *AOC1*-*AOC4* genes, within which *AOC1/AOC2* and *AOC3/AOC4* formed separate subgroups that implied distinct subtype divergence (Figure 1). Notably, the *AOC* genes from allotetraploid cotton species clustered closely with their respective homologs from A-genome and D-genome progenitor species, which reflected the dual-origin nature of the *AOC* gene family in polyploid cotton. The results demonstrated that the diversification of *Gossypium AOC* genes occurred prior to speciation events within the genus. Among cotton species, genetic distances separating distinct *AOC* subtypes were substantially greater, while *AOC* gene members displayed considerable evolutionary conservation within the *Gossypium* genus.

Systematic analyses of conserved motifs, functional domains, and gene structural features of *Gossypium AOC* genes were performed in the context of their phylogenetic relationships (Figure S2A), with members within the same clade displaying highly consistent motif distribution and composition patterns. Most AOC proteins contained the core conserved motifs of the family (Motif 3, Motif 4, Motif 5, and Motif 6) (Figure S2B). All AOC proteins harbored a complete and highly conserved AOC superfamily domain, underscoring their functional conservation (Figure S2C). Gene structure analysis further uncovered that exon arrangement and number were highly conserved among family members, indicating structural stability of the coding region (Figure S2D). In contrast, sequence variation was primarily observed in the length of untranslated regions (UTRs), with distinct differences among *AOC* subtypes and *Gossypium* species.

### 2.3. Interspecific Collinearity Evaluation of the AOC Genes in Cotton

**Figure 2:**
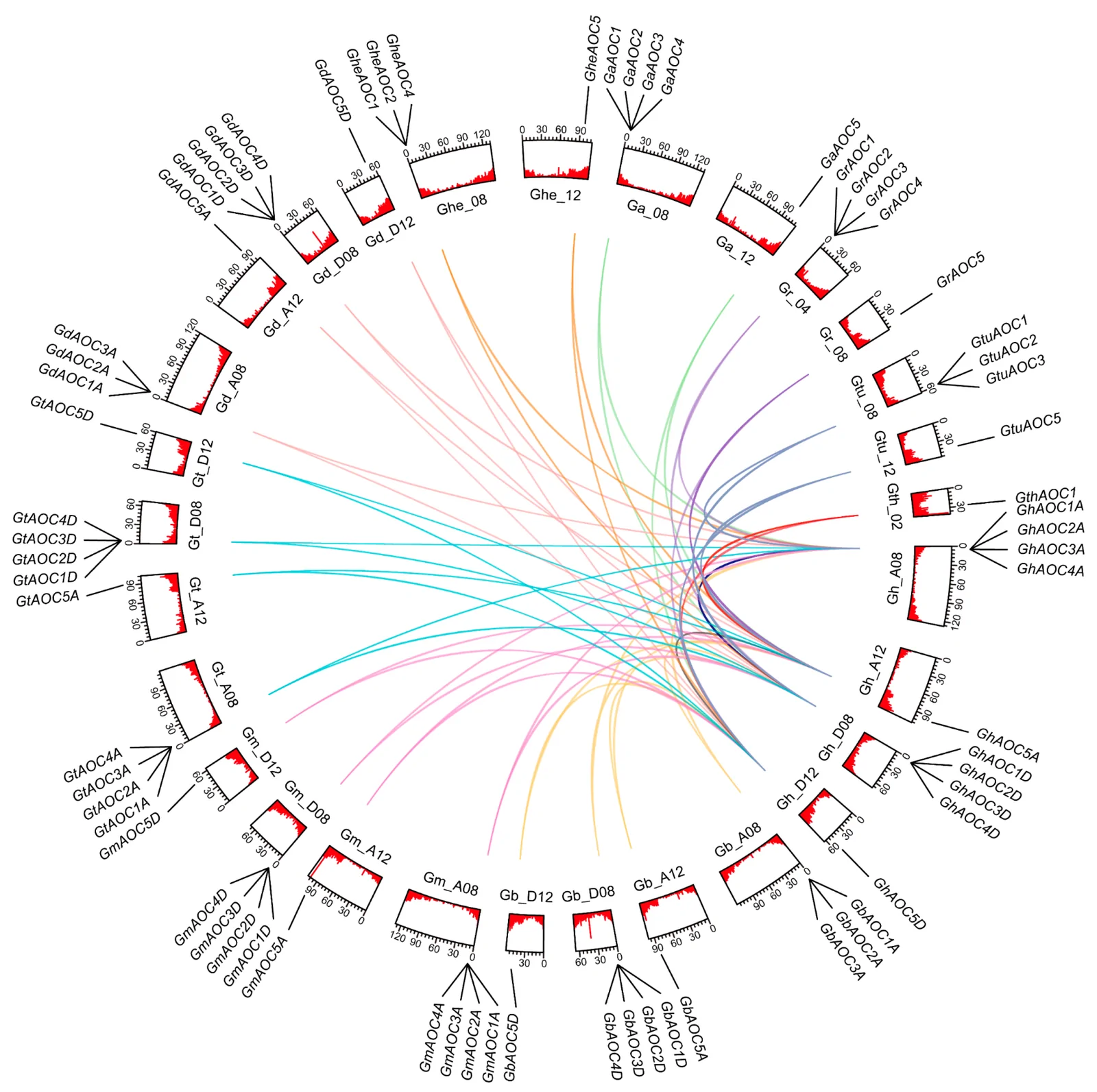
Collinearity analysis of *AOC* genes in *G. hirsutum* and other nine *Gossypium* species. Colored lines denote the orthologous collinear gene pairs among different species. Red bars represent the gene density in corresponding chromosomal regions.

To elucidate the evolutionary pattern and expansion dynamics of *AOC* family members in depth, synteny analysis was performed using homologous gene pairs between *G. hirsutum* and nine additional *Gossypium* species (Figure 2). Our findings demonstrated that *GhAOCs* exhibited a comparable number of collinear gene pairs with *AOC* genes from both diploid and allotetraploid cotton species, with 4 collinear gene pairs identified for *GheAOCs*, *GaAOCs*, *GthAOCs*, and *GdAOCs*, and 2 for *GrAOCs*. In contrast, *GhAOCs* shared 8 collinear gene pairs with *AOCs* from each of the four other allotetraploid species (*GbAOCs*, *GtAOCs*, *GmAOCs*, and *GdAOCs*). These syntenic relationships indicated that the *AOC* gene family experienced expansion during polyploidization and speciation events while retaining high sequence conservation.

### 2.4. Cis-Acting Element Analysis of AOC Gene Promoters in Cotton

**Figure 3:**
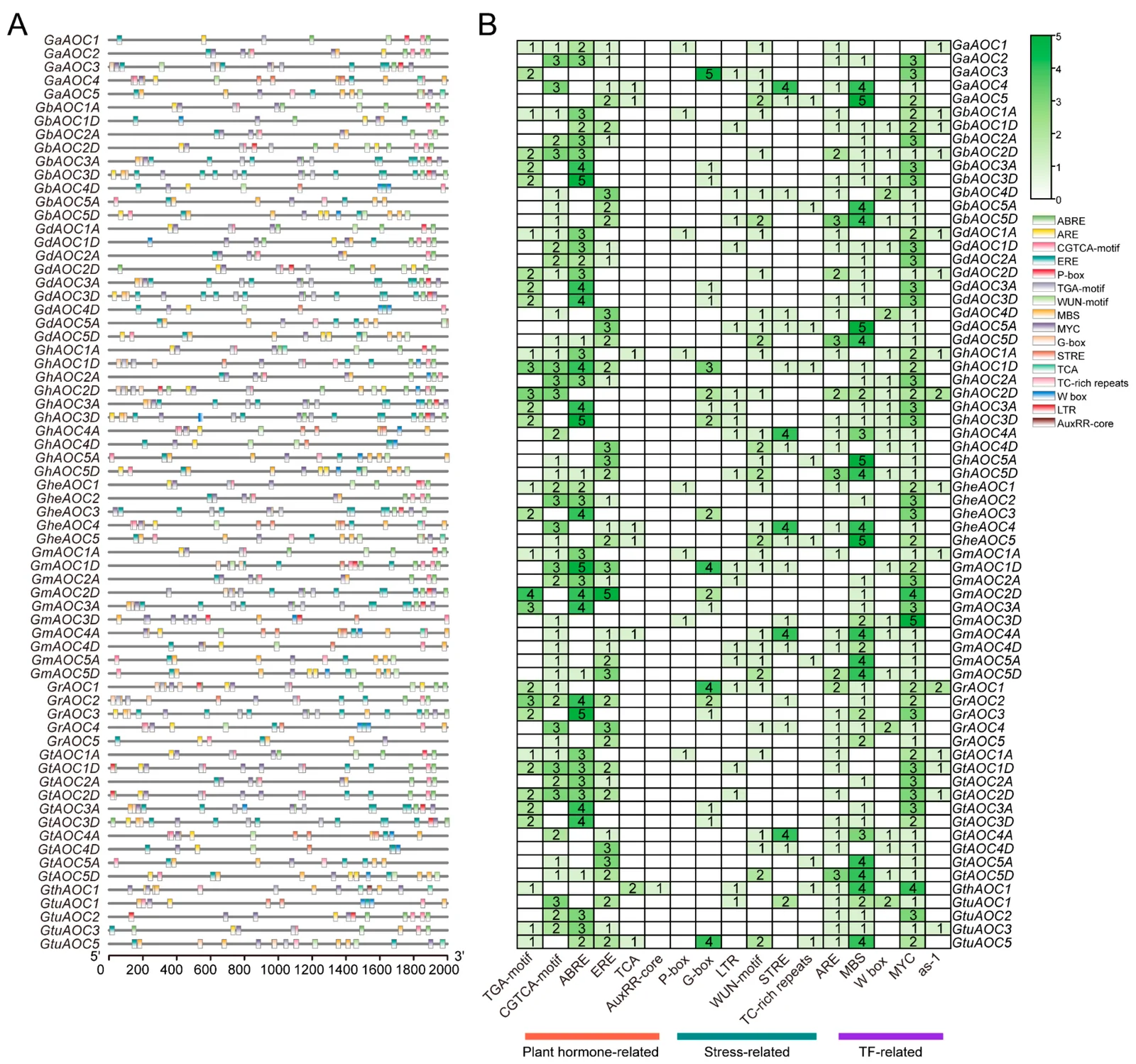
Distribution and abundance of the cis-acting regulatory elements in the promoter regions of *AOC* genes across 10 *Gossypium* species. (**A**) *Cis*-regulatory elements of the 2000-bp promoter sequences of 68 *AOC* genes. Different categories of *cis*-elements are presented with colored boxes. (**B**) Heatmap of the quantity of various *cis*-elements in the *AOC* promoters. Relative abundance of *cis*-elements is depicted by color intensity. These *cis*-elements are grouped into three major classes according to functional annotation: plant hormone-related, stress-related, and TF-related.

To explore the transcriptional regulatory mechanism and potential function of *AOC* genes, *cis*-acting regulatory elements distributed on the 2000-bp promoter sequences of the 68 cotton *AOC* genes were predicted and analyzed (Figure 3). The results indicated that all *AOC* promoters contained abundant and diverse *cis*-acting elements, falling into three major functional groups: plant hormone-related elements, stress-responsive elements, and transcription factor (TF)-binding elements. Regarding distribution patterns, the overall composition of these elements was largely conserved among different *AOC* promoters, whereas the copy number of individual element types exhibited substantial variation. Notably, abscisic acid-responsive elements (ABRE), JA-responsive TGACG-motifs, and ethylene-responsive elements (ERE) were widely distributed with high frequency, and the *cis*-acting elements linked to abiotic stress were prominently enriched, including drought-responsive elements (MBS, ABRE), low-temperature-responsive elements (LTR), and wound-responsive WUN-motif. Additionally, multiple transcription factor binding sites such as MBS (MYB binding site) and W-box were distinctly spotted across the promoters. These results revealed that the transcriptional expression of *Gossypium AOCs* was subject to complex, coordinated regulation by environmental stress, endogenous hormones, and upstream transcription factor networks, indicating the potential role of *AOCs* in abiotic stress response and hormone signal transduction.

### 2.5. Expression Profiling of the AOC Gene Members in Upland Cotton

**Figure 4:**
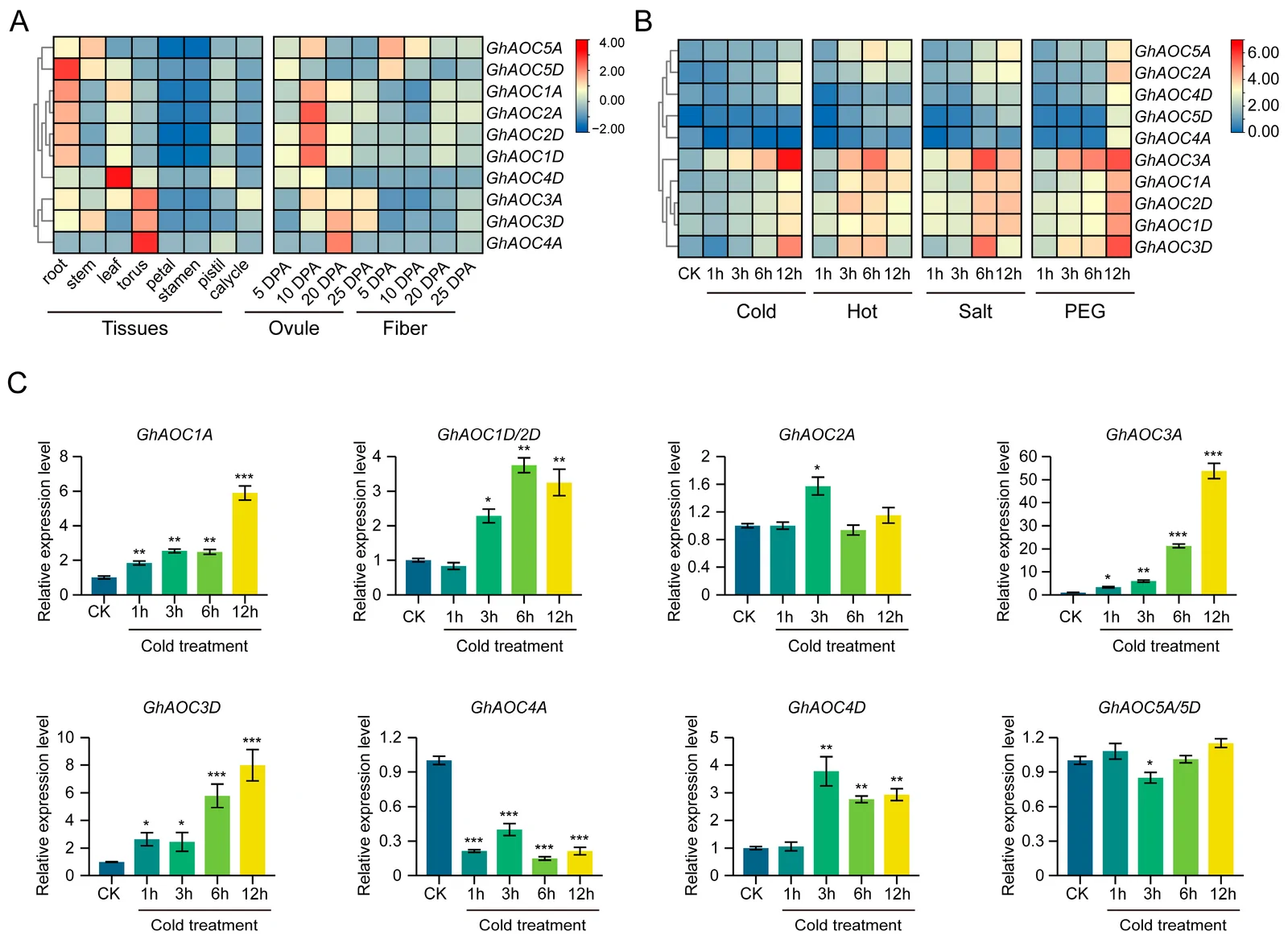
Expression patterns of *AOC* genes in upland cotton. (**A**) Heatmap of expression patterns of *GhAOC* genes across a variety of tissues (root, stem, leaf, torus, petal, stamen, pistil, calycle) and ovule and fiber developmental phases. (**B**) Heatmap of expression of *GhAOC* genes under various abiotic stresses such as chill, heat, salt, and PEG-mediated drought. Heatmap values are presented as log_2_(FPKM + 1); blue, yellow, and red denote low, intermediate, and high levels of expression, respectively. (**C**) qRT-PCR analysis of *GhAOCs* expression under cold treatment (4 °C) at different time points. Error bars are shown with standard deviation (SD, *n* = 3). Statistical significance was generated by One-way ANOVA combined with Tukey’s post-hoc test, with *, **, and *** representing significant differences at *p* < 0.05, 0.01, and 0.001 levels, respectively.

Public transcriptome datasets (PRJN248163) were employed to evaluate the biological function of *AOCs* by systematically profiling their spatiotemporal expression patterns across diverse tissues, during ovule and fiber development, and under multiple abiotic stress conditions. *GhAOCs* appeared to have distinct tissue-specific expression patterns, showing significant expression of *GhAOC1A/1D*, *GhAOC2A/2D*, and *GhAOC5D* in roots, *GhAOC4D* in leaves, and *GhAOC3A/3D* and *GhAOC4A* in torus (Figure 4A). Most *GhAOCs* exhibited high expression levels during ovule development, whereas *GhAOC5A/5D* were specifically implicated in early fiber elongation. Under abiotic stress conditions, *GhAOCs* showed broad expression responses to cold, heat, salt, and drought treatments (Figure 4B). Interestingly, *GhAOC3A* demonstrated continuously upregulated expression across all four stress conditions, with the most pronounced accumulation in response to chilling stress. Quantitative validation further confirmed that *GhAOC3A* served as a key member involved in chilling stress responses (Figure 4C). Together, these outcomes implied that *GhAOC3A* acted as a key component that contributes to the chilling response network in cotton.

### 2.6. GhAOC3A Localized to Chloroplasts and Was Associated with JA Metabolism

**Figure 5:**
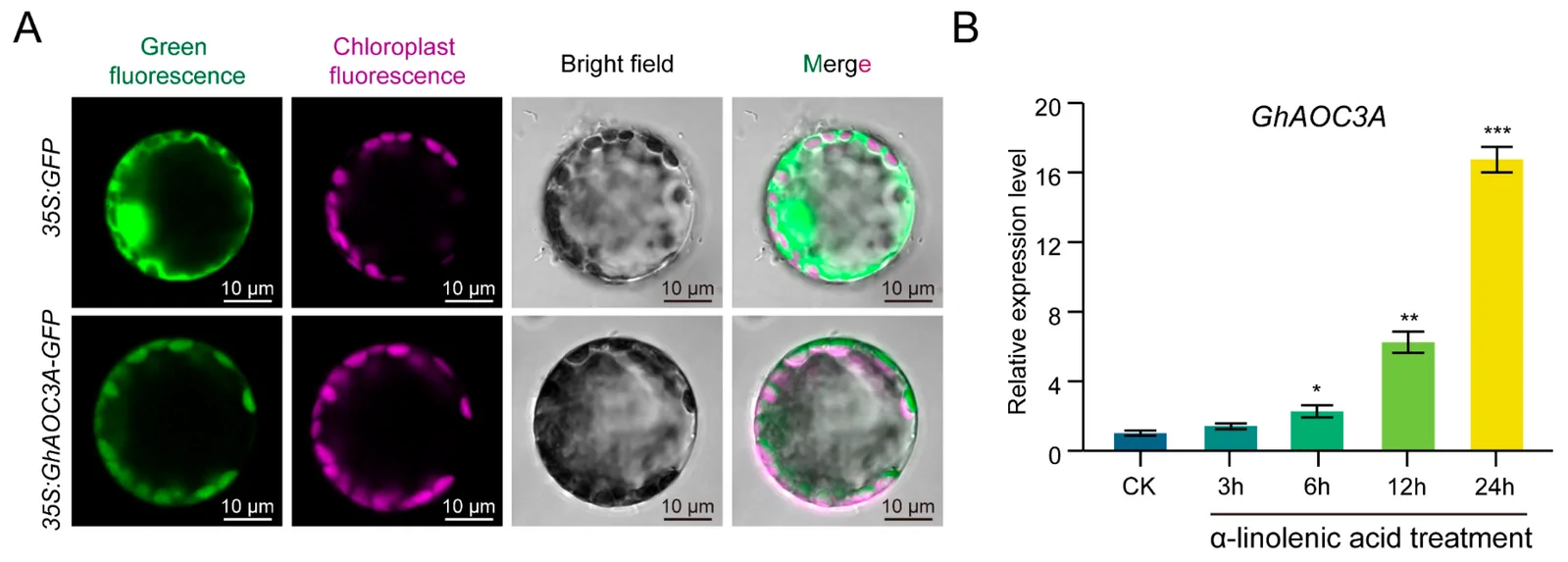
GhAOC3A localizes to chloroplasts and is associated with JA metabolism. (**A**) Subcellular localization analysis of GhAOC3A protein. Images show GFP fluorescence, chloroplast autofluorescence, bright-field views, and merged signals, respectively. Scale bar = 10 µm. (**B**) Expression analysis of *GhAOC3A* in cotton treated with α-linolenic acid at different time points. Error bars are displayed with SD (*n* = 3). Statistical significance was determined by One-way ANOVA followed by Tukey’s post-hoc test, with *, **, and *** representing significant differences at *p* < 0.05, 0.01, and 0.001 levels, respectively.

To delineate the subcellular localization of GhAOC3A and decipher its potential function in the JA metabolic pathway, a transient expression assay was carried out in *Nicotiana benthamiana* protoplasts. The outcomes indicated that the green fluorescence signal from the empty vector control *35S: GFP* was distributed throughout both the nucleus and cytoplasm, while the signal from the *35S: GhAOC3A-GFP* evidently overlapped with chloroplast autofluorescence, thus validating the specific localization of GhAOC3A to chloroplasts (Figure 5A). This subcellular distribution was congruent with that of key enzymes involved in JA biosynthesis. α-Linolenic acid was the initial substrate for biosynthesis and could directly modulate the transcriptional induction of JA metabolic pathway genes, and exogenous α-linolenic acid treatment further demonstrated that *GhAOC3A* expression was significantly induced (Figure 5B), with transcript abundance increasing in a time-dependent manner and reaching a maximum at 24 h. Collectively, the results illustrated that *GhAOC3A* might exert its biological functions within chloroplasts where it may contribute to the α-linolenic acid-dependent JA metabolic pathway in cotton.

### 2.7. Overexpression of GhAOC3A Enhanced the Chilling Tolerance in Cotton

**Figure 6:**
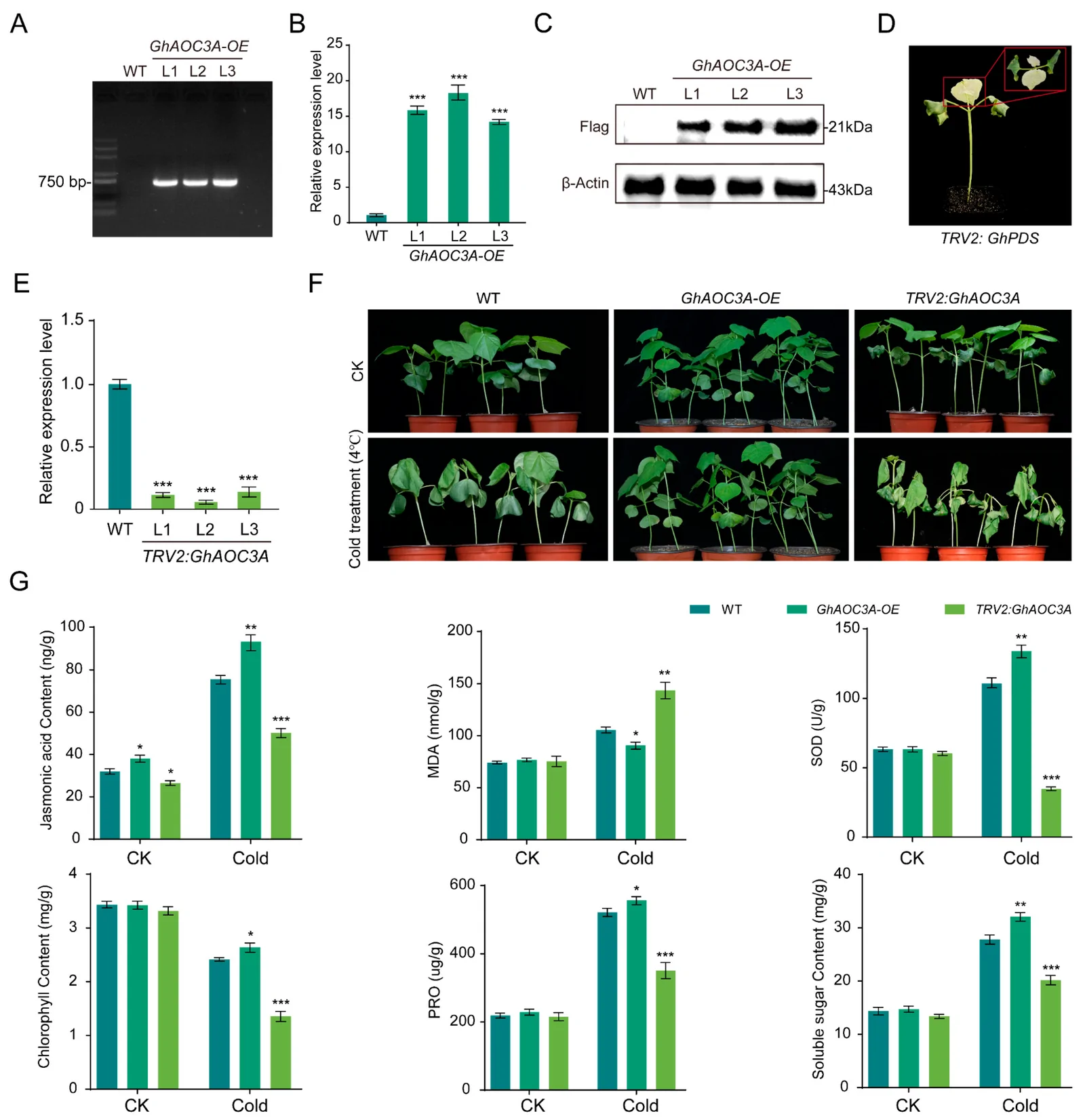
*GhAOC3A* enhanced the chilling tolerance in cotton. (**A**) Electrophoretic images at the DNA level of *GhAOC3A-OE* transgenic cotton. (**B**) Identification of *GhAOC3A* expression levels in *GhAOC3A-OE* cotton plants via qRT-PCR. (**C**) Detection of GhAOC3A-Flag fusion protein expression in *GhAOC3A-OE* transgenic cotton using Western blot (WB). (**D**) Phenotype of cotton plants via VIGS-mediated silencing of *GhPDS*. (**E**) Identification of *GhAOC3A* silencing efficiency in *TRV2:GhAOC3A* cotton plants via qRT-PCR. (**F**) Phenotype analysis of WT, *GhAOC3A-OE*, and *TRV2:GhAOC3A* cotton plants after chilling treatment (48 h); CK: normal temperature, 28 °C. (**G**) Measurements of physiological indices in WT, *GhAOC3A-OE*, and *TRV2:GhAOC3A* cotton plants under chilling conditions; CK: normal temperature, 28 °C. Error bars correspond to the standard deviation (SD) of three biological replicates (*n* = 3). One-way ANOVA and two-way ANOVA both combined with Tukey’s post-hoc multiple comparison test were adopted for statistical analysis. Symbols *, ** and *** signify significant differences at the significance thresholds of *p* < 0.05, 0.01, and <0.001, respectively.

To investigate the genetic role of *GhAOC3A* in the cotton’s response to chilling stress, genetic transformation and virus-induced gene silencing (VIGS) were utilized to create *GhAOC3A*-overexpressing and *GhAOC3A*-silenced cotton plant lines (*GhAOC3A-OE* and *TRV2:GhAOC3A*), with the wild-type (WT) plants as controls. We detected the effectiveness of *GhAOC3A-OE* cotton at the DNA, transcriptional, and translational levels, which confirmed the validity of the *GhAOC3A-OE* transgenic lines (Figure 6A–C). Approximately 10 days post-VIGS infiltration, leaves of the positive control (*TRV2: GhPDS*) displayed a distinct photobleaching phenotype characterized by albino and chlorotic lesions (Figure 6D), confirming successful establishment of the VIGS system and efficient silencing of the target gene. The silencing efficiency was further evaluated via qRT-PCR, which indicated that the transcript levels of *GhAOC3A* were considerably reduced in *TRV2:GhAOC3A* plants relative to the WT (Figure 6E), verifying the effective silencing of *GhAOC3A*. To assess *GhAOC3A* involvement in chilling tolerance, *GhAOC3A-OE*, *TRV2:GhAOC3A* plants, and the control groups were subjected to chilling conditions (4 °C) for 48 h. *GhAOC3A-OE* plants exhibited markedly alleviated chilling injury, with limited leaf wilting, dehydration, and curling under cold treatment (Figure 6F), while *TRV2:GhAOC3A* plants displayed more severe chilling damage relative to WT. These phenotypic differences provided evidence supporting the conclusion that *GhAOC3A* expression markedly influenced cotton chilling tolerance. Meanwhile, physiological and biochemical analyses indicated that *GhAOC3A-OE* plants exhibited increased superoxide dismutase (SOD) activity, which enhanced reactive oxygen species (ROS) scavenging capacity; they also had strengthened osmotic adjustment capacity, which was reflected by elevated proline and soluble sugar contents. Moreover, *GhAOC3A-OE* plants showed reduced malondialdehyde (MDA) accumulation and chlorophyll degradation, which indicated slight membrane lipid peroxidation. Results from VIGS assays provided further supportive evidence for this conclusion. In *TRV2:GhAOC3A* plants, SOD activity was significantly suppressed, leading to compromised ROS scavenging capability. Osmotic adjustment capacity was also impaired, evidenced by decreased proline and soluble sugar contents. Noticeably increased MDA levels and notably reduced chlorophyll contents indicated enhanced membrane lipid peroxidation (Figure 6G). Meanwhile, changes in JA levels were observed from ELISA assays using *GhAOC3A-OE* and *TRV2:GhAOC3A* plants. In summary, overexpression of *GhAOC3A* significantly increased JA accumulation, enhanced antioxidant capacity and osmotic homeostasis and ultimately improved cotton chilling tolerance.

### 2.8. GhWRKY65 Increased the Chilling Tolerance in Cotton by Transcriptionally Activating GhAOC3A Expression

**Figure 7:**
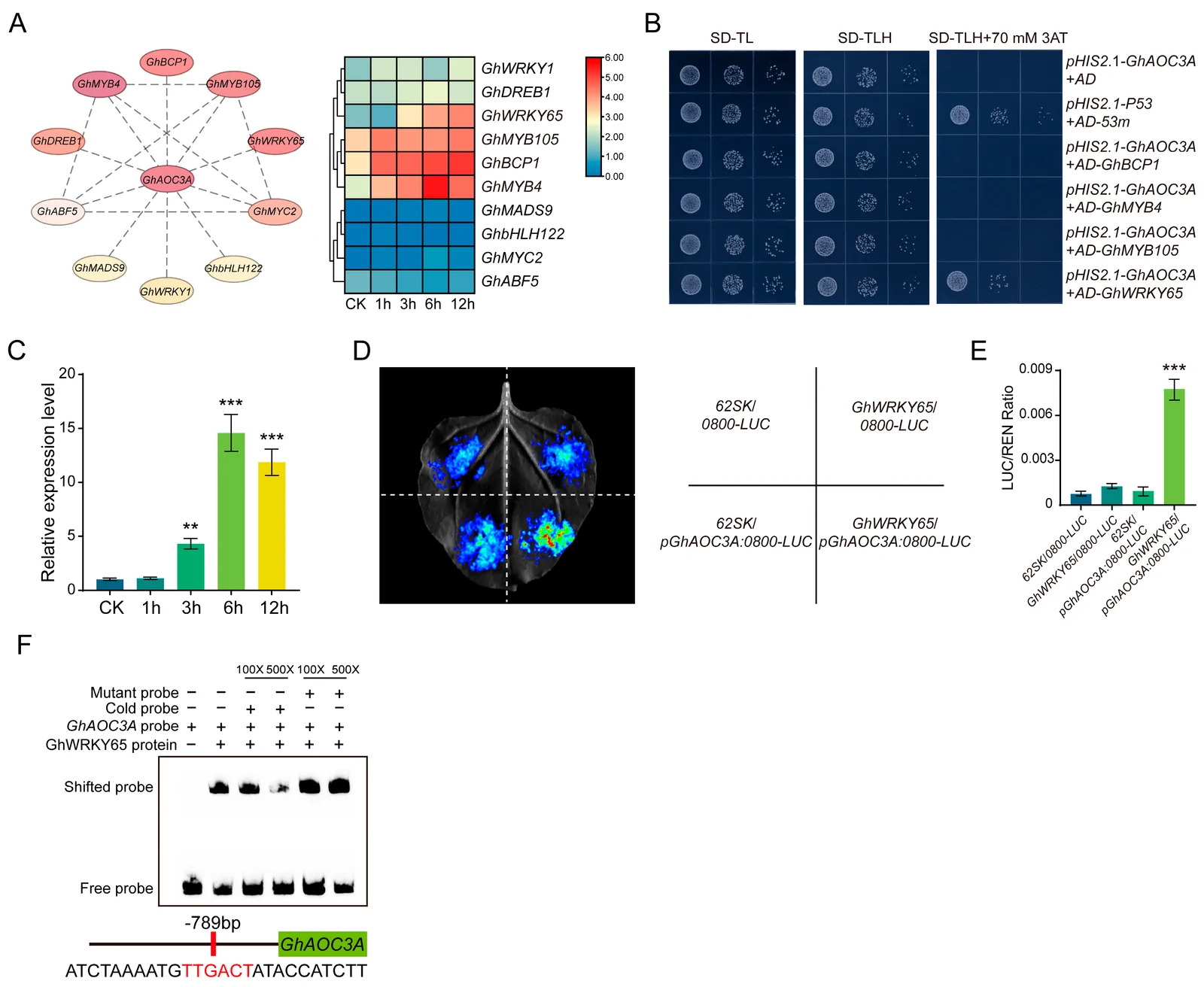
GhWRKY65 increased the chilling tolerance in cotton via transcriptionally activating *GhAOC3A* expression. (**A**) Expression heatmap of transcription factors (TFs), with predicted co-expression regulatory network. (**B**) Y1H verification of interaction between *GhAOC3A* promoter and the candidate TFs. (**C**) qRT-PCR analysis of *GhWRKY65* expression under chilling treatment at a variety of time points. (**D**) Interactive validation of *GhAOC3A* promoter and GhWRKY65 via dual-luciferase reporter assays. (**E**) Quantitative analysis of LUC/REN ratios of *GhAOC3A* promoter and GhWRKY65. (**F**) EMSA analysis of direct binding of GhWRKY65 and the W-box motif (TTGACT) within *GhAOC3A* promoter. Excess unlabeled probes were supplemented to perform competitive binding assays, whereas mutant probes harbored targeted base substitutions within the conserved core motif of the W-box sequence. Standard deviation (SD) values derived from three independent biological replicates (*n* = 3) are presented as error bars. Statistical comparisons are implemented via one-way ANOVA, followed by Tukey’s post-hoc test. Markers ** and *** separately represent statistical significance at *p* < 0.01 and 0.001 levels.

To investigate the upstream transcriptional regulation of *GhAOC3A*, the co-expression module in the CottonMD database was utilized to predict TFs co-expressed with this gene, and four candidate TFs with the highest relevance scores were selected for subsequent validation by yeast one-hybrid (Y1H) assay (Figure 7A). Y1H analysis demonstrated that the TF GhWRKY65 directly interacted with the *GhAOC3A* promoter (Figure 7B). qRT-PCR detections demonstrated that *GhWRKY65* expression was rapidly upregulated in response to a chilling environment, reaching maximum transcript accumulation 6 h after chilling treatment, indicating its potential role in regulating the chilling response (Figure 7C). Furthermore, dual-luciferase reporter (LUC) assays showed that GhWRKY65 substantially improved *GhAOC3A* promoter activity and acted as a positive transcriptional activator, as evidenced by a remarkable increase in luminescence driven by the *GhAOC3A* promoter (Figure 7D,E). Electrophoretic mobility shift (EMSA) assays further validated that GhWRKY65 precisely bound to the W-box element in the *GhAOC3A* promoter in vitro (Figure 7F). When the concentration of unlabeled competitor probes increased, the binding signal generated by biotin-labeled probes and GhWRKY65 gradually diminished, while mutant probes failed to compete effectively for GhWRKY65 binding. These results indicated that GhWRKY65 directly targets and activates *GhAOC3A* transcription by specifically binding to the W-box motif in its promoter region.

**Figure 8:**
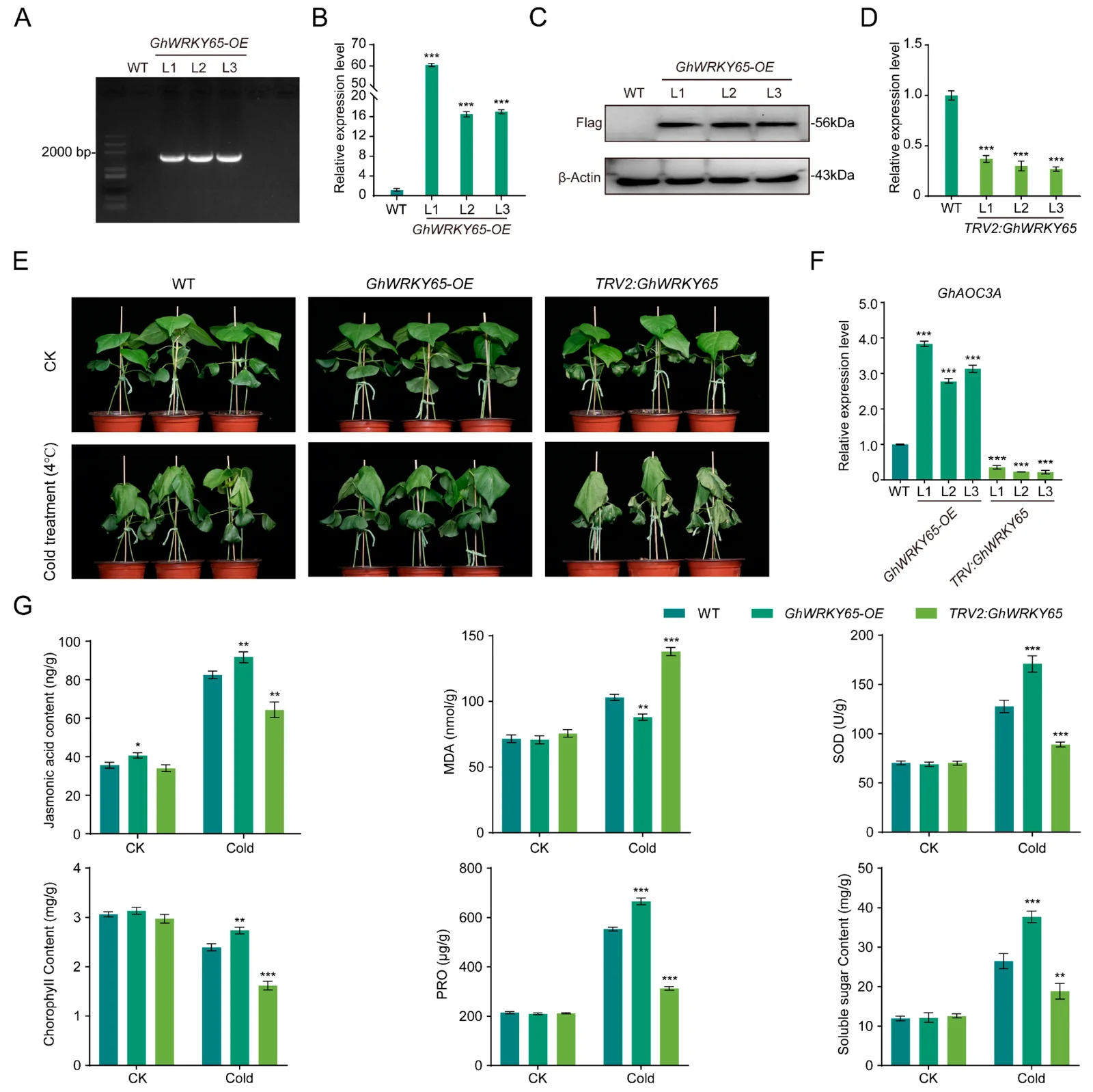
*GhWRKY65* increased the chilling tolerance in cotton. (**A**) Electrophoretic images at the DNA level of *GhWRKY65-OE* transgenic cotton. (**B**) Identification of *GhWRKY65* expression levels in *GhWRKY65-OE* cotton plants via qRT-PCR. (**C**) Detection of GhWRKY65-Flag fusion protein expression in *GhWRKY65-OE* transgenic cotton by using WB. (**D**) Identification of *GhWRKY65* silencing efficiency in *TRV2:GhWRKY65* cotton plants via qRT-PCR. (**E**) Phenotype analysis of WT, *GhWRKY65-OE,* and *TRV2:GhWRKY65* cotton plants under chilling treatment (48 h). (**F**) Relative transcription expression levels of *GhAOC3A* in *GhWRKY65-OE* and *TRV2:GhWRKY65* plants after 6 h of chilling exposure. (**G**) Determination of endogenous JA contents in leaves of WT, *GhWRKY65-OE*, and *TRV2:GhWRKY65* plants during 4 °C and normal conditions (28 °C). Standard deviation (SD) values derived from three biological replicates (*n* = 3) are presented as error bars. Statistical comparisons were implemented via two-way ANOVA, followed by Tukey’s post-hoc test. Markers *, ** and *** separately represent statistical significance at *p* < 0.05, 0.01 and 0.001 levels.

To assess the genetic role of *GhWRKY65* in modulating cotton chilling tolerance, *GhWRKY65-OE* lines and *GhWRKY65*-silenced cotton plants (*TRV2:GhWRKY65*) with high overexpression/silencing efficiency, along with WT, were then exposed to 4 °C cold treatment (Figure 8E). The *35S: GhWRKY65* construct, which was confirmed to be integrated into the genome of *GhWRKY65-OE* lines, could be stably translated into the GhWRKY65-Flag fusion protein (Figure 8A–C). Significantly suppressed expression of *GhWRKY65* in *TRV2: GhWRKY65* plants was detected (Figure 8D). After 48 h of low-temperature exposure, compared with WT, *GhWRKY65-OE* plants exhibited milder cold-damage phenotypes; in contrast, *TRV2: GhWRKY65* plants displayed visible leaf wilting and withering (Figure 8E,G). Additional qRT-PCR analysis showed that *GhAOC3A* transcript abundance was upregulated in *GhWRKY65-OE* plants but markedly downregulated in *TRV2: GhWRKY65* plants (Figure 8F), providing a direct regulatory link between *GhWRKY65* and *GhAOC3A*. Consistent with the results for *GhAOC3A*, we found that *GhWRKY65-OE* lines, which exhibited enhanced SOD activity, improved the antioxidant and osmotic adjustment capacities of cotton, alleviated membrane damage, maintained photosynthesis, and elevated JA levels. In contrast, the opposite was observed in *TRV2: GhWRKY65* cotton plants (Figure 8G). Taken together, these findings suggested that *GhWRKY65* contributed to cotton chilling tolerance and JA accumulation, at least in part, by activating *GhAOC3A* expression.

## 3. Discussion

Low-temperature stress severely impairs cotton growth, posing a chief challenge to sustainable cotton cultivation and production, particularly in high-latitude regions [2]. Accumulating evidence indicates that the phytohormone JA plays an essential role in controlling plant responses to abiotic stress, and AOC has been established as the vital enzyme for JA biosynthesis and for sustaining the proper functionality of JA signal transduction pathways [31,32]. Currently, systematic genome-wide analysis and functional studies of *AOC* genes in *Gossypium* species remain insufficiently investigated. In our investigation, we systematically identified 68 *AOC* gene members across 10 distinct *Gossypium* species, with notable divergence in gene copy numbers among these species, showing that allotetraploid cotton species typically harbor a greater number of *AOC* genes than their diploid counterparts (Figure S1). This distribution pattern aligns with the reports of *AOC* gene expansion in other polyploid plants, implying that whole-genome duplication events played an important role in expanding the *AOC* gene family in cotton [33]. Analogous contraction events have been documented in other plant lineages and are often linked to species-specific adaptive processes, implying potential functional specialization of *AOC* genes in *G. thurberi* [34]. Chromosomal mapping of *Gossypium AOC* genes revealed a combination of evolutionary conservation and divergence, with *AOCs* in allotetraploid species displaying symmetric distribution between the A and D subgenomes and consistent chromosomal localizations (Figure S1). Among these chromosomes, *AOCs* on A08 and D08 are more densely populated and evolutionarily conserved, which aligns with previously described genomic stability in cotton [35]. The findings provide additional evidence for the widely accepted model in which allotetraploid cotton arose through interspecific hybridization and genome doubling involving A- and D-genome diploid ancestors [36]. Phylogenetic analysis clustered the *AOCs* into two major clades, where members within each clade display conserved motif compositions and retain intact *AOC* superfamily domains (Figure 1). This is consistent with the conserved nature of *AOCs* documented in other plants, while divergence between the two clades suggests functional differentiation, a common evolutionary feature of gene families [37].

The promoters of *AOCs* contained *cis*-acting elements linked to hormone signaling and environmental stress, including elements responsive to JA, ABA, and ETH (Figure 3), which is consistent with the coordinated regulation of these phytohormones in stress environments [38]. Moreover, the enrichment of low-temperature and other abiotic stress-responsive elements in AOC promoters strongly corroborates their important role in plant environmental adaptation [39]. Given the extensive crosstalk among diverse phytohormone signaling pathways [40,41], these findings provided additional evidence at the transcriptional regulatory level that *AOC* genes may function as core nodes integrating hormonal cues and stress signals. Expression profiling further highlighted the functional divergence among *AOC* family members, with distinct spatial expression patterns observed across different tissues (Figure 4). Most *AOCs* were highly expressed during ovule development, whereas *GhAOC5A/D* were preferentially upregulated during early fiber elongation. This observation was consistent with reports showing that JA contributes to cotton fiber development [42] and highlights significant roles of *AOCs* in plant growth and development. Notably, *GhAOC3A* was markedly induced under multiple abiotic stresses, with the most significant accumulation observed under low-temperature treatment. This pattern resembles the functional characteristics of JA biosynthesis-related genes in other plants [43]. GhAOC3A is targeted to chloroplasts (Figure 5A), which is consistent with the chloroplast localization of other enzymes responsible for JA biosynthesis [44]. Exogenous application of α-linolenic acid significantly upregulated *GhAOC3A* expression (Figure 5B), supporting the notion that *GhAOC3A* responds to upstream JA precursors and is associated with JA-related responses.

Cold stress prominently disrupts plant physiological processes and metabolic homeostasis [45], as low temperature reduces membrane fluidity and impairs membrane integrity, thereby increasing electrolyte leakage. Furthermore, excessive ROS accumulation induces oxidative damage, prompting plants to trigger antioxidant defenses by elevating the activities of antioxidant enzymes such as SOD [46]. Consistent with these physiological responses, our data revealed that chilling stress activates antioxidant defense in cotton seedlings (Figure 6G). If the antioxidant system fails to maintain redox balance, enhanced lipid peroxidation and elevated MDA levels will occur [5]. Low temperature also perturbs osmotic homeostasis by altering the accumulation of proteins, proline, and soluble sugars, thereby compromising cellular water balance. Photosynthesis is also highly sensitive to low temperature, with reduced chlorophyll content and diminished photosynthetic efficiency serving as key indicators [5]. Furthermore, our physiological measurements confirmed that chilling stress altered the abundance of osmoprotectants and chlorophyll in cotton seedlings (Figure 6G). GmAOC3 is a key enzyme that regulates JA biosynthesis in soybean, and *GmAOC3-OE* lines exhibit significantly elevated endogenous JA levels [18]. Transgenic analyses of tomato sense and antisense *AOC* lines revealed that altered *AOC* expression modulates endogenous JA accumulation and enhances wound-induced defense signaling [47]. Our molecular assays showed that *GhAOC3A* expression levels were correlated with changes in JA abundance under chilling stress (Figure 6G). It should be noted that these JA measurements were obtained using an ELISA kit with limited specificity; thus, the observed differences in JA abundance represent apparent changes rather than definitive evidence for altered JA biosynthetic or metabolic flux. A growing body of literature has established the critical functions of *AOC* in mediating plant adaptations to abiotic and biotic stresses. Overexpression of *MfAOC2* activated the antioxidant system and improved plant tolerance to chilling environments. In tomato, upregulation of *AOC* genes promotes JA biosynthesis, which enhances plant drought adaptability. In this study, functional analyses based primarily on overexpression materials indicated that *GhAOC3A* plays an essential part in cotton chilling tolerance. *GhAOC3A-OE* cotton showed enhanced chilling tolerance. VIGS results provided further supportive evidence for this function, while we acknowledge the inherent limitations of the VIGS system (Figure 6F). Our results further reinforced the known involvement of JA accumulation in plant stress responses and supported the notion that *GhAOC3A* may participate in cotton chilling adaptation linked to JA-related metabolism.

**Figure 9:**
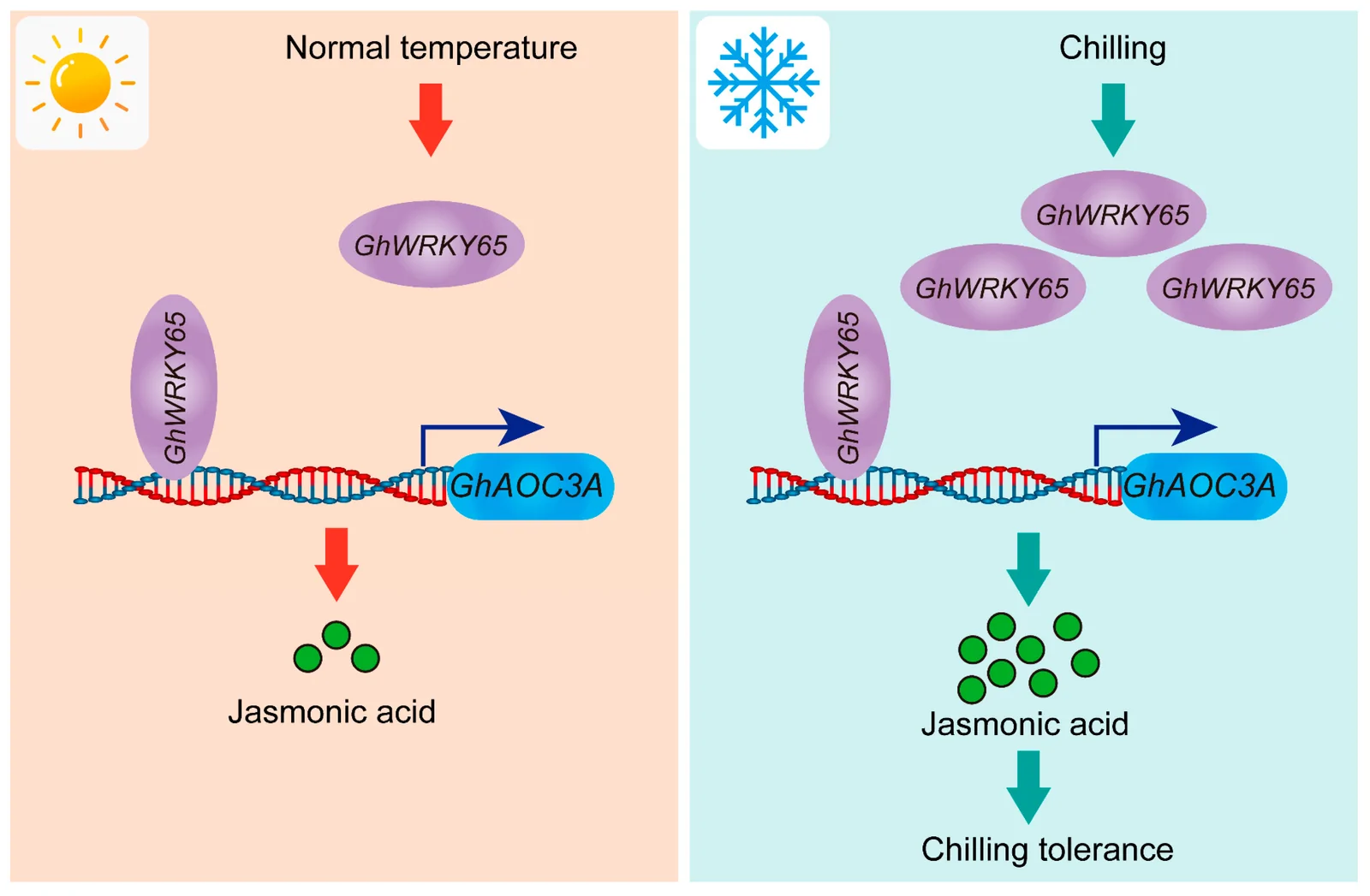
Model of the GhWRKY65–*GhAOC3A* module as one regulatory pathway for chilling tolerance in cotton.

Transcription factors act as core modulators orchestrating the hierarchical regulatory networks governing plant adaptive responses to environmental stresses. Extensive research has validated the tight functional linkage between WRKY family proteins and diverse abiotic stress signaling cascades [48]. In the present study, multiple biochemical and genetic assays confirmed the direct physical association between GhWRKY65 and the W-box motifs within the *GhAOC3A* promoter and demonstrated that GhWRKY65 binding induces transcriptional activation of *GhAOC3A* (Figure 7B,D,F). This discovery may expand the existing framework describing WRKY-dependent control over the JA metabolic pathway (Figure 7). In tomato, the SlWRKY42-*SlMYC2* regulatory cascade triggers the transcriptional activation of JA biosynthetic genes, driving endogenous JA buildup and reinforcing salt stress resilience in this crop species [49]. For apple, physical interactions between MdWRKY9 and two JAZ repressors, MdJAZ5 and MdJAZ10, coordinate salt acclimation via transcriptional tuning of genes responsible for JA metabolic flux. As the consensus *cis*-motif exclusively targeted by WRKY transcription factors, the W-box harbors the invariant core pentanucleotide TTGACC/T and occurs abundantly across promoter sequences in terrestrial plant genomes [50,51]. Members of the WRKY superfamily also exert indispensable functions during plant acclimation to chilling stimuli. In rice, OsWRKY76 elevates freezing tolerance through modulation of the *DREB1B*-mediated signal cascade, whereas CiWRKY31 positively modulates cold adaptive capacity in citrus plants [27,50]. Consistent with these reports, phenotypic and physiological data from *GhWRKY65* overexpression lines support the positive role of *GhWRKY65* in chilling tolerance, and VIGS results provide additional supportive evidence (Figure 8E,G). Furthermore, based on ELISA measurements, we observed apparent changes in JA abundance in *GhWRKY65-OE* and *TRV2:GhWRKY65* plants. Collectively, these phenotypic and molecular data supported a model in which *GhWRKY65* enhanced plant chilling tolerance, possibly by activating *GhAOC3A* expression and contributing to alterations in JA-associated responses (Figure 9). This echoes earlier mechanistic reports documenting transcription factor-directed stress adaptation through downstream effector genes, highlighting the central importance of transcriptional reprogramming for plant survival under chilling environments [52].

Overall, our results confirmed that GhWRKY65 positively regulated *GhAOC3A* transcription, thereby establishing a novel GhWRKY65–*GhAOC3A* regulatory module involved in cotton responses to chilling stress. These findings deepen the mechanistic understanding of transcriptional modulation governing plant *AOC* family genes and shed light on the adaptive mechanisms associated with cotton chilling tolerance. Future studies will further expand the mechanistic depth of the GhWRKY65–*GhAOC3A* module. First, owing to the intrinsic limitations of the ELISA assay, other compounds in plant crude extracts may interfere with JA quantification. Future work will employ LC-MS for more precise quantification of specific metabolites within the JA metabolic pathway. Second, transcriptomic and metabolomic studies on plants with the GhWRKY65–*GhAOC3A* module will advance mechanistic understanding of intermediate metabolites and gene alterations that occur in the JA biosynthetic pathway. Finally, given the limitations of VIGS and the potential effects of viral infection, future generations of gene-knockout lines will enable a more accurate evaluation of this module’s functional potential.

## 4. Materials and Methods

### 4.1. Plant Materials and Cold Treatments

The cotton samples employed for the research were all sourced from *Gossypium hirsutum* (TM-1). The cotton seedlings were subjected to growth chambers with precise climate control functions, where a photoperiod of 16 h light and 8 h dark cycle was maintained at a constant 28 °C ambient temperature. *Nicotiana benthamiana* was cultivated at 22 °C under a similar light/dark cycle setting. The leaf tissues were harvested at 1, 3, 6, and 12 h post-treatment for relative expression analysis via qRT-PCR. All harvested leaf tissues were quickly submerged in liquid nitrogen and preserved at −80 °C for subsequent analysis. We chilled four-week-old cotton seedlings (OE/VIGS) at 4 °C, while control seedlings were maintained at 28 °C. We carried out all treatments in climate-controlled growth chambers at 55% relative humidity under the photoperiod described above.

### 4.2. Genome-Wide Identification of the AOC Gene Members in Cotton

The CottonMD [53] was utilized to retrieve genome sequences for various *Gossypium* species including diploid species of *Gossypium arboreum* (CRI, A2), *G. herbaceum* (WHU, A1), *G. raimondii* (NSF, D5), *G. thurberi* (ISU, D1), and *G. turneri* (NSF, D10), as well as allotetraploid species of *G. darwinii* (HGS, AD5), *G. mustelinum* (JGI, AD4), *G. tomentosum* (HGS, AD3), *G. barbadense* (H7124, AD2), and *G. hirsutum* (JGI, AD1). The recognized *AOC* family members of *Arabidopsis thaliana* were acquired from the TAIR [54], and their protein sequences were queried against the protein datasets of the 10 *Gossypium* species using local BLASTP (v2.17.0) to identify homologous sequences. To exclude false-positive identifications, an E-value (≤1 × 10^−20^) cutoff was implemented, and the remaining parameters were all maintained at their default state. We extracted genomic coordinates and position details for the detected *AOC* genes from the corresponding GFF3 annotation files using the “GFF3/GTF Gene Position Parse” function implemented in TBtools (v2.390) [55]. Then the MG2C (v2.1, Huazhong Agricultural University, Wuhan, China) online tool was employed to map *AOC* family members across chromosomes in 10 cotton genomes. *AOC* genes were named according to a combination of chromosomal location, gene number, and species abbreviation. Physicochemical qualities of the encoded AOC proteins were calculated employing the “Protein Parameter Calc” module in TBtools-II (v2.390, South China Agricultural University, Guangzhou, China), including instability index, isoelectric point, and molecular weight.

### 4.3. Phylogenetic Analysis of the AOC Gene Members

The AOC protein sequences from 10 *Gossypium* species were aligned using the ClustalW algorithm available in MEGA (v12.0, Temple University, Philadelphia, PA, USA) with default parameters [56]. Subsequently, the evolutionary relationships among the *AOC* genes across various species were deduced through a phylogenetic tree created employing the Neighbor-Joining (NJ) technique with 2000 bootstrap repetitions. The resultant tree was then visualized and refined utilizing iTOL, an online interactive software (v7.0, Biobyte Solutions GmbH & EMBL, Heidelberg, Germany).

### 4.4. Gene Structure and Promoter Assessment of the AOC Gene Members

The conserved motifs of AOC proteins across various *Gossypium* species were analyzed by MEME Suite (v5.5.9, The University of Queensland, Brisbane, Australia), under the following configured parameters: a maximum number of motifs = 6, a motif width restricted to 6–100 amino acids, and a ZOOPS distribution model, with all other settings maintained at default values. To further validate the identified *AOC* family members, the Conserved Domain Database (CDD) hosted by the National Center for Biotechnology Information (NCBI) was employed to confirm conserved functional domains. To explore the transcriptional regulatory features of *AOC* genes in cotton, the 2000-bp upstream sequences were extracted and analyzed using the PlantCARE (Ghent University, Ghent, Belgium) platform to predict *cis*-acting elements. The Gene Structure View (Advanced) module of TBtools was then utilized to visualize all gene structure and motif distribution data.

### 4.5. Interspecies Synteny Analysis of the AOC Genes

A detailed interspecies synteny investigation across the 10 cotton genomes was carried out using the One Step MCScanX (Pure Java) within TBtools. The Advanced Circos toolkit (v2.467) was applied to visualize the detected collinear associations of the *AOC* genes.

### 4.6. Quantitative Real-Time PCR (qRT-PCR) Analysis

For the qRT-PCR assay, we used fresh treated cotton tissues or samples stored at −70 °C, which were ground in an RNase-free environment to prevent extraneous RNA interference; total RNA isolated from cotton tissues was then examined via electrophoresis to evaluate RNA quality. After integrity and purity analysis, the EasyScript cDNA Synthesis SuperMix (TransGen, Beijing, China) was utilized to select the extracted RNA for the cDNA synthesis. The cDNA samples from different treatment groups, which were diluted to a uniform working concentration with sterile water, could be stored briefly at −20 °C after preparation. Then, a qRT-PCR assay was carried out using SYBR Green-based premix (TIANGEN Biotech, Beijing, China) on a LightCycler 480 Real-Time PCR System (Roche Biotech, Mannheim, Germany) with *GhUBQ7* as an internal control. All gene-specific primer sequences used in our investigation are presented in Supplementary Table S2.

### 4.7. Subcellular Localization of GhAOC3A Protein

The complete coding sequence (CDS) of *GhAOC3A* was PCR-amplified and subsequently integrated into the *pSYB03: GFP* (*35S: GFP*) expression vector utilizing the homologous recombination method, thus generating the fusion vector *pSYB03-GhAOC3A: GFP* (*35S:GhAOC3A: GFP*). The resulting plasmid construct was then transformed into protoplasts isolated from 2-week-old leaf tissues of *Nicotiana benthamiana*, followed by incubation in darkness at 25 °C for 24 h. Preliminary morphological screening of isolated protoplasts was performed via light microscopy, whereby morphologically intact, spherical protoplasts were singled out for subsequent fluorescence detection. Laser scanning confocal microscopy (Nikon, Tokyo, Japan) was applied to capture fluorescent signals, with excitation and emission wavelengths configured as follows: 640 nm/675 nm for intrinsic chloroplast autofluorescence, and 488 nm/510 nm for green fluorescent protein (GFP). Parallel transformations with the empty *pSYB03-EGFP* vector were conducted to serve as the negative control group.

### 4.8. Generation of Overexpressing and VIGS-Silenced Cotton Plants

We successfully constructed the *35S: GhAOC3A-Flag* and *35S: GhWRKY65-Flag* overexpression vectors. When the OD value of the *Agrobacterium* (EHA105) culture reached 0.9, the embryonic tips of TM-1 cotton were infected via an ultrasound-assisted infiltration method. They were co-cultured on the medium for three days. Then the embryonic tips were placed upright on spectinomycin-containing MS selection medium, and we subcultured every two weeks for 2 to 3 successive subcultures. Explants with resistant axillary buds were transferred to 1/2 MS medium for root induction. More than three independent lines were obtained after spectinomycin screening. We used the stably inherited T1 generation plants for subsequent experiments. To generate specific VIGS fragments, the complete CDSs of *GhAOC3A* and *GhWRKY65* were uploaded to the CottonGVD database (v1.0, Institute of Cotton Research, CAAS, Anyang, China), following previously described methods [57]. High-scoring, gene-unique conserved regions were chosen for the construction of silencing vectors. Briefly, 150-bp target fragments were amplified using the CDSs of *GhAOC3A* and *GhWRKY65* as templates. The recovered amplicons were ligated into the *TRV2* vector. *TRV1* was used as the helper plasmid for viral replication [58]. The *TRV1* and engineered *TRV2* constructs were independently mixed and co-infiltrated into *Agrobacterium tumefaciens* (GV3101) by syringe-mediated infiltration. Mixed agrobacterial cultures (OD = 1.2) were prepared as an infiltration suspension, which was then infiltrated into cotyledon tissues of 2-week-old TM-1 cotton seedlings. Infiltrated seedlings were maintained in complete darkness for 24 h prior to transferring to standard growth chambers. 14 days after infiltration, qRT-PCR analysis was performed to assess the transcriptional knockdown efficiency of target genes in individual silenced cotton lines.

### 4.9. Determination of Physiological Indices in Chilling Stress

We selected representative cotton plants (with moderate overexpression or silencing efficiency) from the overexpression lines and VIGS plants for physiological index measurements. Multiple individual cotton plants were available within the representative overexpression lines (*GhAOC3A-OE* L1 and *GhWRKY65-OE* L2). One seedling was selected from each of three independent cold-treatment batches for measurement. For VIGS plants, one seedling was chosen from each of three independent cold-treatment batches. These three individual seedlings served as the three biological replicates for physiological measurements. We used the average technical replicate value for each sample in the analysis. The experimental unit for statistical analysis was an individual cotton seedling. The collected leaf samples from different treatment groups with or without cold-stress exposure were ground and homogenized under an ice-cold environment using a homogenizer. The homogenates were subsequently subjected to physiological index measurements, including malondialdehyde (MDA) concentration, superoxide dismutase (SOD) enzymatic activity, chlorophyll content, proline (PRO) accumulation, and soluble sugar levels, with detection performed using matching biochemical assay kits (Product Nos. BC6410, BC0170, BC0990, BC0290, BC0030; Solarbio, Beijing, China). Endogenous JA content was measured employing a commercial ELISA kit (Product No. SP29738, Spbio, Wuhan, China). Briefly, 1 g of cotton leaf tissue was weighed, added to 9 mL of PBS, and homogenized. 1 mL of the resulting supernatant was aspirated and diluted 10-fold. After a 10-fold dilution of the extract, sample loading, incubation, washing, and color development were carried out according to the manufacturer’s instructions. A standard curve was constructed using serially diluted JA standards. All physiological measurements were normalized per gram fresh weight (FW) of leaf samples.

### 4.10. Yeast One-Hybrid Assay (Y1H)

Using homologous recombination, the complete CDS of the candidate transcription factor (TF) was inserted into the *pGADT7* (*AD*) vector. In addition, the promoter sequence of *GhAOC3A* was inserted into the *Phis2.1* vector. The co-transformed yeast cells were screened on selective media containing varying concentrations of 3-amino-1,2,4-triazole (3AT) to verify the optimal inhibitory concentration. Then, the interaction verification between the TF and its target promoter was conducted at an optimal 3AT concentration, with the plates being kept for 72 h at 30 °C.

### 4.11. Dual-Luciferase Reporter Assay

The full-length coding sequence of *GhWRKY65* and the promoter sequence of *GhAOC3A* were separately inserted into the *pGreenII 62SK* and *pGreenII 0800-LUC* vectors. After vector construction, Sanger sequencing was performed, and sequence-verified plasmids were preserved. After transforming A. tumefaciens (GV3101) with the two recombinant plasmids, the agrobacterial suspensions were infiltrated into young *N. benthamiana* leaves. Afterward, the activities of Renilla luciferase (REN) and firefly luciferase (LUC) were subsequently computed employing a Dual-Luciferase Reporter Assay System (Coolaber, Beijing, China).

### 4.12. Electrophoretic Mobility Shift Assay (EMSA)

The CDS of *GhWRKY65* was PCR-amplified and inserted into *pET28a* to generate the *GhWRKY65–His* recombinant expression vector, producing GhWRKY65 protein fused with a C-terminal histidine tag. The construct was transformed into DE3-competent cells for heterologous protein induction and expression. For EMSA, 5′-biotin-labeled DNA probes were used, while unlabeled wild-type probes were served as cold competitors and prepared without biotin modification. In vitro protein–DNA binding interactions were verified using a Chemiluminescent EMSA Kit (Beyotime, Shanghai, China). All probe sequences synthesized for EMSA are provided in Supplementary Table S2.

### 4.13. Statistical Analysis and Visualization

GraphPad Prism (v10.4.0, GraphPad Software Biotech, Boston, MA, USA) software was utilized for dataset processing, statistical calculation, and figure visualization. One-way Analysis of Variance (ANOVA) was utilized to compare the differences across numerous experimental groups under single-factor treatments, and two-way ANOVA was performed to dissect the interactive effects of two independent variables, with significance levels indicated as *p* < 0.05, *p* < 0.01, and *p* < 0.001, respectively.

## 5. Conclusions

In conclusion, this research performed systematic genome-wide identification and characterization of *AOC* family genes in 10 cotton species, yielding a total of 68 validated *AOC* gene members. *GhAOC3A* served as a pivotal positive regulator of cotton chilling tolerance and JA accumulation. GhWRKY65 acted as an upstream activator of *GhAOC3A* transcription by binding to the *GhAOC3A* promoter. Our findings suggested an association between the GhWRKY65-*GhAOC3A* module and chilling stress responses in cotton. These findings enriched the current mechanistic framework of cotton chilling stress adaptation and highlighted key genetic targets for the development of chilling-tolerant cotton germplasms.

## Data Availability

Data are contained within the article and Supplementary Materials. Further data presented in this study are available upon request from the corresponding author.

## Supplementary Materials

The following supporting information can be downloaded at: https://www.mdpi.com/article/10.3390/plants15213417/s1, Figure S1: Chromosomal localization of AOC gene members in ten Gossypium species. (A) *G. hirsutum*; (B) *G. barbadense*; (C) *G. tomentosum*; (D) *G. mustelinum*; (E) *G. darwinii*; (F) *G. arboretum*; (G) *G. raimondii*; (H) *G. herbaceum*; (I) *G. turneri*; (J) *G. thurberi*. The scale to the left of the chromosome depicts its length (Mb), and gene names are labeled at their corresponding positions on the chromosome; Figure S2: Conserved motifs, protein domains, and gene structure analysis of the *AOC* genes in *Gossypium* species. (A) Phylogenetic tree of *AOC* genes. (B) Conserved motif distribution of AOC proteins. (C) Conserved domain distribution of the AOC superfamily. (D) Exon-intron structure of *AOC* genes. Different colors represent distinct conserved motifs, CDS and UTR elements indicated in the legend; Table S1: Analysis of the physical and chemical properties of *AOC* genes; Table S2: The primers used in this study.
