# Supplementary material for: The GhWRKY65–GhAOC3A Module Enhances the Chilling Tolerance in Cotton by Regulating Jasmonic Acid Accumulation
Source: Plants (Basel). 2026 Nov 3;15(21):3417. doi: 10.3390/plants15213417 (PMC13766131; doi:10.3390/plants15213417)
Supplement: Supplementary file 1 [file plants-15-03417-s001.zip › plants-4649078-supplementary.pdf]

Supplementary Figures and Tables

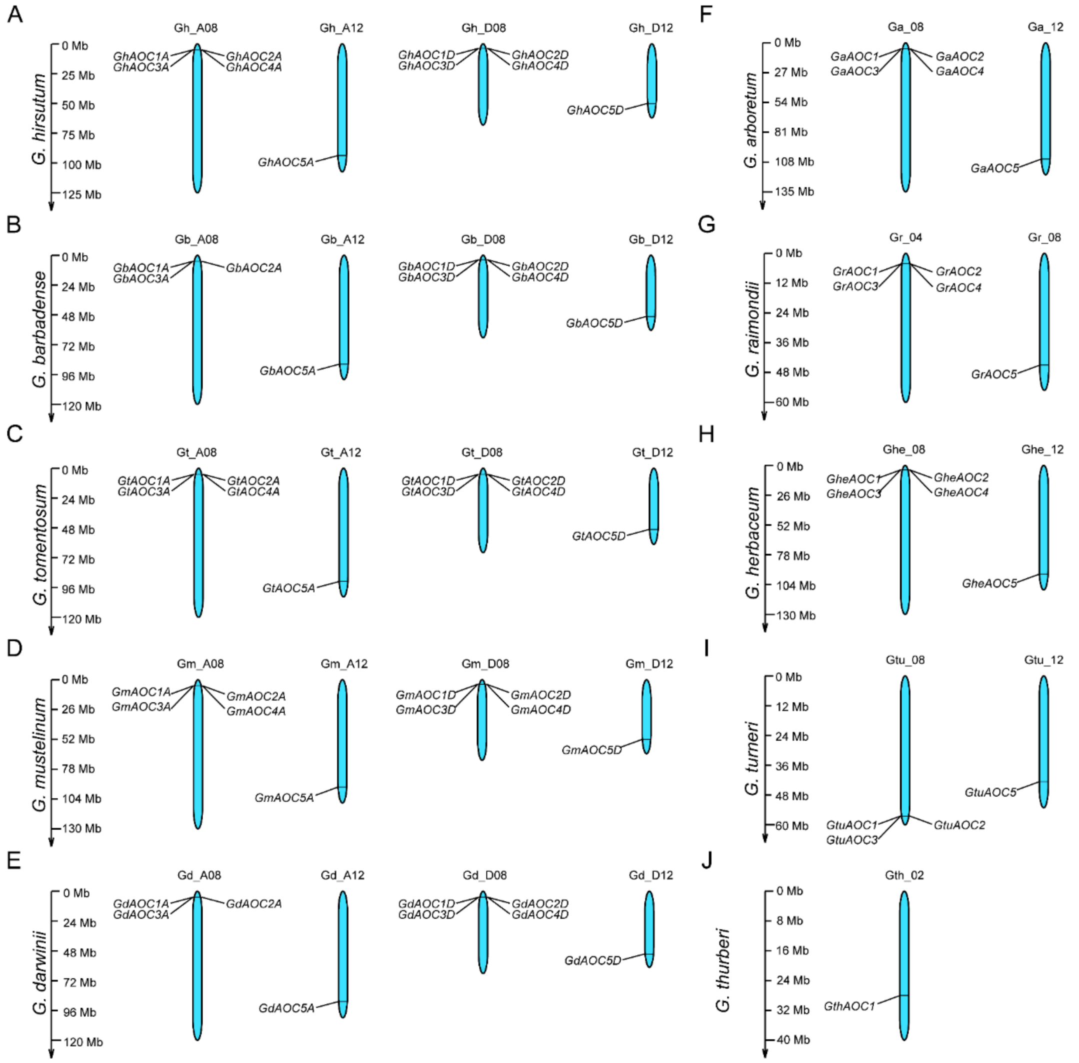

Figure S1. Chromosomal localization of AOC gene members in ten Gossypium species. (A) *G. hirsutum*; (B) *G. barbadense*; (C) *G. tomentosum*; (D) *G. mustelinum*; (E) *G. darwinii*; (F) *G. arboretum*; (G) *G. raimondii*; (H) *G. herbaceum*; (I) *G. turneri*; (J) *G. thurberi*. The scale to the left of the chromosome depicts its length (Mb), and gene names are labeled at their corresponding positions on the chromosome.

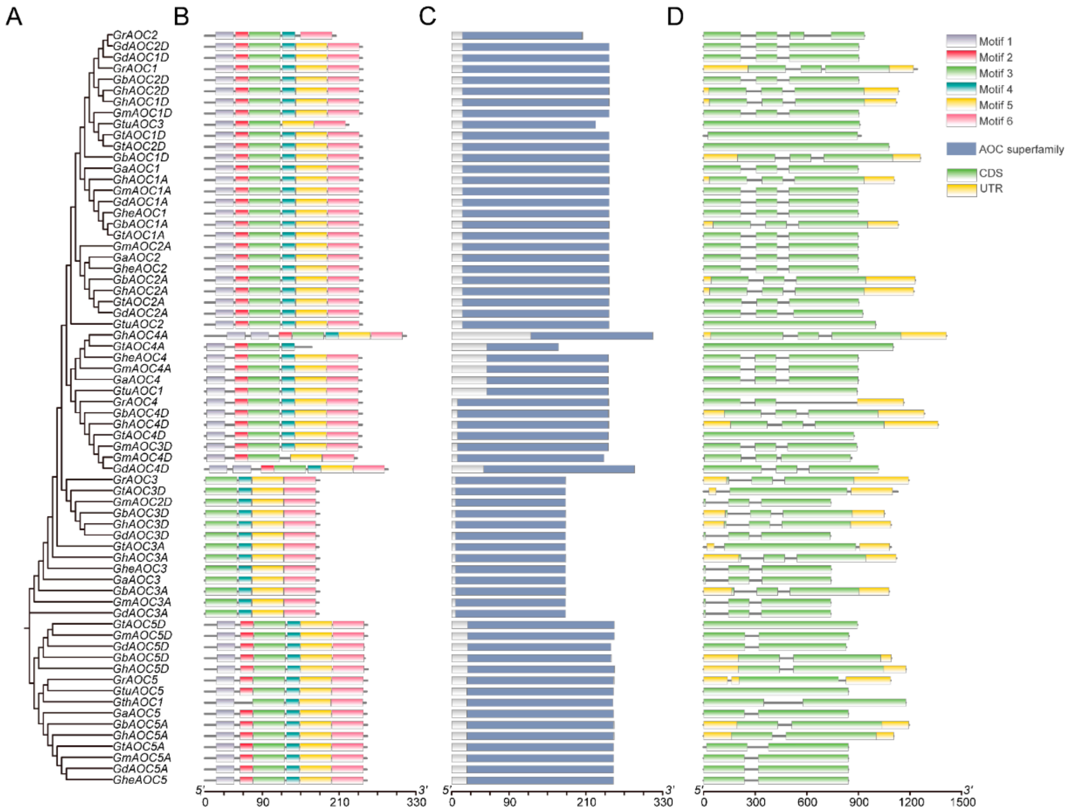

Figure S2. Conserved motifs, protein domains, and gene structure analysis of the *AOC* genes in *Gossypium* species. (A) Phylogenetic tree of *AOC* genes. (B) Conserved motif distribution of AOC proteins. (C) Conserved domain distribution of the AOC superfamily. (D) Exon-intron structure of *AOC* genes. Different colors represent distinct conserved motifs, CDS and UTR elements indicated in the legend.

Table S1. Analysis of the physical and chemical properties of *AOC* genes

Gene Name	Gene ID	Length of protein (AA)	Molecular Weight (kDa)	Theoretical PI	Instability Index	Aliphatic Index	Grand Average of Hydropathicity
GaAOC1	*Gar08G04410*	246	27.03	8.72	43.49	83.7	-0.185
GaAOC2	*Gar08G04420*	246	27.06	8.7	45.72	83.29	-0.218
GaAOC3	*Gar08G04430*	178	19.76	5.85	37.19	87.7	-0.107
GaAOC4	*Gar08G04440*	245	27.13	8.77	43.92	80.49	-0.188
GaAOC5	*Gar12G22200*	253	27.85	8.95	40.34	73.24	-0.292
GbAOC1A	*Gobar.A08G041500*	246	27.01	8.52	43.49	85.28	-0.151
GbAOC1D	*Gobar.D08G044400*	246	27.07	8.51	44.24	81.71	-0.212
GbAOC2A	*Gobar.A08G041600*	246	27.04	8.7	46.5	81.71	-0.24
GbAOC2D	*Gobar.D08G044500*	246	27.07	8.51	44.24	81.71	-0.212
GbAOC3A	*Gobar.A08G041700*	178	19.79	5.85	36.35	87.7	-0.122
GbAOC3D	*Gobar.D08G044600*	178	19.75	5.85	34.79	86.07	-0.144
GbAOC4D	*Gobar.D08G044700*	245	27.02	9.06	46.87	81.31	-0.161
GbAOC5A	*Gobar.A12G178800*	253	27.75	8.95	41.44	73.24	-0.292
GbAOC5D	*Gobar.D12G191700*	249	27.31	9.1	40.21	74.82	-0.251
GdAOC1A	*Godar.A08G044300*	246	27.03	8.72	43.49	83.7	-0.185
GdAOC1D	*Godar.D08G049200*	246	27.07	8.51	44.24	81.71	-0.212
GdAOC2A	*Godar.A08G044400*	246	27.04	8.7	46.5	81.71	-0.24
GdAOC2D	*Godar.D08G049300*	246	27.07	8.51	44.24	81.71	-0.212
GdAOC3A	*Godar.A08G044500*	178	19.79	5.85	36.35	87.7	-0.122
GdAOC3D	*Godar.D08G049400*	178	19.75	5.85	34.79	86.07	-0.144
GdAOC4D	*Godar.D08G049500*	286	31.71	9.16	46.6	75.45	-0.22
GdAOC5A	*Godar.A12G194800*	253	27.75	8.95	41.44	73.24	-0.292
GdAOC5D	*Godar.D12G202400*	249	27.31	9.1	40.21	74.82	-0.251
GhAOC1A	*Gohir.A08G036200*	246	27.03	8.72	43.49	83.7	-0.185
GhAOC1D	*Gohir.D08G046900*	246	27.10	8.51	44.7	81.71	-0.214
GhAOC2A	*Gohir.A08G036300*	246	27.04	8.7	46.5	81.71	-0.24
GhAOC2D	*Gohir.D08G047000*	246	27.10	8.51	44.7	81.71	-0.214
GhAOC3A	*Gohir.A08G036400*	178	19.81	5.85	36.35	85.51	-0.133
GhAOC3D	*Gohir.D08G047100*	178	19.75	5.85	34.79	86.07	-0.144
GhAOC4A	*Gohir.A08G036500*	314	35.26	9.3	42.33	76.15	-0.224
GhAOC4D	*Gohir.D08G047200*	245	27.02	9.06	46.87	81.31	-0.161
GhAOC5A	*Gohir.A12G169800*	253	27.75	8.95	41.44	73.24	-0.292
GhAOC5D	*Gohir.D12G173100*	254	27.87	8.95	40.72	72.95	-0.293
GheAOC1	*Ghe08G04400*	246	27.03	8.72	43.49	83.7	-0.185
GheAOC2	*Ghe08G04410*	246	27.07	8.86	47.29	83.29	-0.223
GheAOC3	*Ghe08G04420*	178	19.79	5.95	38.51	87.13	-0.128
GheAOC4	*Ghe08G04430*	245	27.13	8.77	43.92	80.49	-0.188
GheAOC5	*Ghe12G22110*	253	27.75	8.95	41.44	73.24	-0.292
GmAOC1A	*Gomus.A08G044200*	246	27.03	8.72	43.49	83.7	-0.185

GmAOC1D	*Gomus.D08G046400*	246	27.07	8.51	44.24	81.71	-0.212
GmAOC2A	*Gomus.A08G044300*	246	27.08	8.71	46.57	81.71	-0.242
GmAOC2D	*Gomus.D08G046500*	178	19.76	5.85	36.35	86.63	-0.132
GmAOC3A	*Gomus.A08G044400*	178	19.79	5.85	36.35	87.7	-0.122
GmAOC3D	*Gomus.D08G046600*	245	27.02	9.06	46.87	81.31	-0.161
GmAOC4A	*Gomus.A08G044500*	245	27.13	8.77	43.92	80.49	-0.188
GmAOC4D	*Gomus.D08G046700*	238	26.17	9.18	49.38	80.42	-0.145
GmAOC5A	*Gomus.A12G183100*	253	27.75	8.95	41.44	73.24	-0.292
GmAOC5D	*Gomus.D12G194400*	254	27.84	8.95	40.72	73.35	-0.283
GrAOC1	*Gorai.004G046100*	246	27.07	8.51	44.24	81.71	-0.212
GrAOC2	*Gorai.004G046200*	204	22.39	9.21	49.08	84.17	-0.247
GrAOC3	*Gorai.004G046300*	178	19.76	5.85	36.35	86.63	-0.132
GrAOC4	*Gorai.004G046400*	245	27.03	9.06	45.86	81.71	-0.16
GrAOC5	*Gorai.008G185600*	253	27.76	8.95	40.08	73.24	-0.292
GtAOC1A	*Gotom.A08G045500*	246	27.07	8.88	44.27	82.11	-0.219
GtAOC1D	*Gotom.D08G048300*	246	27.10	8.51	46.02	81.3	-0.226
GtAOC2A	*Gotom.A08G045600*	246	27.04	8.7	46.5	81.71	-0.24
GtAOC2D	*Gotom.D08G048400*	246	27.11	8.51	46.02	82.89	-0.204
GtAOC3A	*Gotom.A08G045700*	178	19.77	5.85	36.35	87.13	-0.12
GtAOC3D	*Gotom.D08G048500*	178	19.76	5.85	36.35	86.63	-0.132
GtAOC4A	*Gotom.A08G045800*	167	18.63	9.54	48.34	76.59	-0.361
GtAOC4D	*Gotom.D08G048600*	245	27.02	9.06	46.87	81.31	-0.161
GtAOC5A	*Gotom.A12G199800*	253	27.75	8.95	41.44	73.24	-0.292
GtAOC5D	*Gotom.D12G202900*	254	27.84	8.95	38.24	73.35	-0.28
GthAOC1	*Gothu.00033756*	252	27.63	8.75	39.61	74.29	-0.316
GtuAOC1	*TURN.08g232250*	245	27.05	9.06	49.1	81.31	-0.17
GtuAOC2	*TURN.08g232260*	246	26.98	8.51	45.28	82.11	-0.197
GtuAOC3	*TURN.08g232270*	225	24.79	8.27	46.1	75.47	-0.288
GtuAOC5	*TURN.02g354040*	253	27.75	8.95	40.42	72.85	-0.293

Table S2. The primers used in this study

Gene_ID	Prime name	Sequence (from 5'to 3')
Gohir.A08G036200	*GhAOC1A-F-QPCR*	TTCTCCCACCCATTCCTTGC
	GhAOC1A-R-QPCR	AGTGAAACCTCGCTTGGGAG
Gohir.D08G046900	*GhAOC1D-F-QPCR*	CTCCCAAGCGAGGTTTCACT
	GhAOC1D-R-QPCR	CGAAGGTAAGCTGGACTCCC
Gohir.A08G036300	*GhAOC2A-F-QPCR*	GTGTCTCCGGTCAGGTCAAG
	GhAOC2A-R-QPCR	TTTGCAATGGTGGCATGTGG
Gohir.D08G047000	*GhAOC2D-F-QPCR*	CTCCCAAGCGAGGTTTCACT
	GhAOC2D-R-QPCR	CGAAGGTAAGCTGGACTCCC
Gohir.A08G036400	*GhAOC3A-F-QPCR*	AAGAGCTGCTTTGCAAACCG
	GhAOC3A-R-QPCR	TGAAATTTGCAATGGCGGCA
Gohir.D08G047100	*GhAOC3D-F-QPCR*	TAAGATTTTCTACACTTTCTAT
	GhAOC3D-R-QPCR	GCCTTAGCACCCGGAGCGGCTT
Gohir.A08G036500	*GhAOC4A-F-QPCR*	TACCAGGACACGTTTCTCGC
	GhAOC4A-R-QPCR	AGCATCCGGAAAGGCTTCAA
Gohir.D08G047200	*GhAOC4D-F-QPCR*	GCAGTGCAGGGACCTTACTT
	GhAOC4D-R-QPCR	CTTCTGGGAGCTCACCGATC
Gohir.A12G169800	*GhAOC5A-F-QPCR*	GCTGGACTCTGTGTGCTGAT
	GhAOC5A-R-QPCR	AATCCCAGATCCTCCCGTCA
Gohir.D12G173100	*GhAOC5D-F-QPCR*	GCTGGACTCTGTGTGCTGAT
	GhAOC5D-R-QPCR	AATCCCAGATCCTCCCGTCA
Gohir.D04G135800	*GhWRKY65-F-QPCR*	CCACAATGTTCCTGCTGCAC
	GhWRKY65-R-QPCR	TTGTTCTGCAGCATCCCCAA
GenBank: DQ116441	*GhUBQ7-F-QPCR*	AAGAAGAAGACCTACACCAAGCC
	GhUBQ7-R-QPCR	GCCCACACTTACCGCAATA
Gohir.A08G036400	*GhAOC3A-F*	ATGGCCGCCTCGGATAAAGTTCAAG
	GhAOC3A-R	ATTAGTGAAATTTGCAATGGCGGCA
Gohir.D04G135800	*GhWRKY65-F*	ATGGCTGCTTCATCATCATCTGCT
	GhWRKY65-R	AGACAGGAATCCGTCCAAAAATGA
Gohir.A08G036400	*GhAOC3A-TRV2-F*	agtggtctctgtccagtcctGTATATGAGATGAATGAAAGAGATC
	GhAOC3A-TRV2-R	ggtctcagcagaccacaagtCATCCCTGCAGTTATTCCTATCCGT
Gohir.D04G135800	*GhWRKY65-TRV2-F*	agtggtctctgtccagtcctATGGCTGCTTCATCATCATCTGCTT
	GhWRKY65-TRV2-R	ggtctcagcagaccacaagtGGTAGGGGAAGGAGCAAATGACTTG
Gohir.A08G036400	*GhAOC3-pHIS2.1-F*	gactcactatagggcgaattcTCAGTCCAATCCAATTCAAATAACA
	GhAOC3-pHIS2.1-R	gcgtgagctccccgggaattcCAGTCTTCACATGTGAACTATTATT
Gohir.D04G135800	*GhWRKY65-AD-F*	gccatggaggccagtgaattcATGGCTGCTTCATCATCATCTGCT

	GhWRKY65-AD-R	cagctcgagctcgatggatccAGACAGGAATCCGTCCAAAAATGA
Gohir.D12G129700	*GhBCP1-AD-F*	gccatggaggccagtgaattcATGCCTGTTGATGCTACTTCGATTA
	GhBCP1-AD-R	cagctcgagctcgatggatccCTTACCCTTTTTTCTTCTTTTGGAA
Gohir.A11G227400	*GhMYB105-AD-F*	gccatggaggccagtgaattcATGAGTCTACCGAATCTAAATAATG
	GhMYB105-AD-R	cagctcgagctcgatggatccAAGAAGCTCCAACTCCAAGAAAATC
Gohir.A12G037700	*GhMYB4-AD-F*	gccatggaggccagtgaattcATGGGAAGGTCTCCTTGCTGTGAGA
	GhMYB4-AD-R	cagctcgagctcgatggatccTTTCATTTCCAAACTTCTGTAATCC
Gohir.A08G036400	*GhAOC3-0800-F*	ttcctgcagcccgggggatccTCAGTCCAATCCAATTCAAATAACA
	GhAOC3-0800-R	ggaagggtcttgcgggatccCAGTCTTCACATGTGAACTATTATT
Gohir.D04G135800	*GhWRKY65-62SK-F*	ccccgggctgcaggaattcATGGCTGCTTCATCATCATCTGCT
	GhWRKY65-62SK-R	ccgggccccccctcgagAGACAGGAATCCGTCCAAAAATGA
Gohir.A08G036400	*GhAOC3-WT-Probe-F*	Biotin-ATCTAAAATGTTGACTATACCATCTT
	GhAOC3-WT-Probe-R	AAGATGGTATAGTCAACATTTTAGAT
Gohir.A08G036400	*GhAOC3-Mutant-Probe-F*	ATCTAAAATGAAAAAAATACCATCTT
	GhAOC3-Mutant-Probe-R	AAGATGGTATTTTTTTCATTTTAGAT
Gohir.A08G036400	*GhAOC3-Competitor-Probe-F*	ATCTAAAATGTTGACTATACCATCTT
	GhAOC3-Competitor-Probe-R	AAGATGGTATAGTCAACATTTTAGAT
